# Multicomponent syntheses of pyrazoles via (3 + 2)-cyclocondensation and (3 + 2)-cycloaddition key steps

**DOI:** 10.3762/bjoc.20.178

**Published:** 2024-08-16

**Authors:** Ignaz Betcke, Alissa C Götzinger, Maryna M Kornet, Thomas J J Müller

**Affiliations:** 1 Heinrich-Heine-Universität Düsseldorf, Math.-Nat. Fakultät, Institut für Organische Chemie und Makromolekulare Chemie, Universitätsstrasse 1, D-40225 Düsseldorf, Germany; 2 Zaporizhzhia National University, Faculty of Biology, Department of Chemistry, Zhukovskogo Street 66, 69600 Zaporizhzhia, Ukraine

**Keywords:** cycloaddition, cyclocondensation, multicomponent reaction, one-pot reactions, pyrazole

## Abstract

Pyrazoles are rarely found in nature but are traditionally used in the agrochemical and pharmaceutical industries, while other areas of use are also actively developing. However, they have also found numerous other applications. The search for new and efficient syntheses of these heterocycles is therefore highly relevant. The modular concept of multicomponent reactions (MCR) has paved a broad alley to heteroaromatics. The advantages over traditional methods are the broader scope and increased efficiency of these reactions. In particular, traditional multistep syntheses of pyrazoles have considerably been extended by MCR. Progress has been made in the cyclocondensation of 1,3-dielectrophiles that are generated in situ. Limitations in the regioselectivity of cyclocondensation with 1,3-dicarbonyls were overcome by the addition–cyclocondensation of α,β-unsaturated ketones. Embedding 1,3-dipolar cycloadditions into a one-pot process has additionally been developed for concise syntheses of pyrazoles. The MCR strategy also allows for concatenating classical condensation-based methodology with modern cross-coupling and radical chemistry, as well as providing versatile synthetic approaches to pyrazoles. This overview summarizes the most important MCR syntheses of pyrazoles based on ring-forming sequences in a flashlight fashion.

## Introduction

Pyrazoles and 1,2-diazoles [1] have received considerable interest in the past years. Although they are rarely found in nature [2], their spectrum of biological activity is remarkably broad, leading to numerous applications in pharmaceutical chemistry [3–6]. For instance, pyrazoles serve as monoamine oxidase A and B inhibitors [7] and as COX-II inhibitors [8], making them valuable analgesics [9]. Furthermore, several blockbuster drugs, such as VIAGRA^®^ [10], Celecoxib^®^ [11], and Rimonabant [12], contain pyrazole cores. In addition, extensive agrochemical uses of pyrazoles [13] include insecticides [14], herbicides [15], and fungicides [16]. Furthermore, they are widely applied for the assembly of supramolecular ensembles [17] and molecular systems capable of photoinduced electron transfer [18]. Due to their pertinent photophysical properties [19], pyrazoles also find applications in OLED technology [20] and optical brighteners [21] in the textile and laundry industry.

The ongoing quest for novel, potent, and efficient syntheses of pyrazoles prompts the search for the “ideal” synthesis [22–23], one that addresses all the synthetic challenges to meet the ecological and economic requirements. This “ideal” synthesis would commence with simple starting materials, progress through safe, catalytic, and quantitative conversions, and culminate in streamlined processes, all conducted within a single reaction vessel. Multicomponent reactions (MCR) closely approach this ideal, representing a reactivity-based concept where reactive functionalities are generated and consumed in each step [24]. MCR can be conducted in a domino, sequential, or consecutive fashion, offering a versatile approach to synthetic design by creating countless new sequences by concatenation of organic elementary steps in a one-pot fashion. Many reviews on general syntheses of pyrazoles have been published [25–38], as well as a few dedicated reviews on MCR synthesis of pyrazole derivatives [39–41]. In sensu stricto, multicomponent methodology demands that all sequences have to be performed in the same reaction vessel; neither intermediate work-up, filtration of byproducts, nor solvent exchange by evaporation falls within the scope of MCR. Therefore, this review aims to present and discuss the concepts of ring-forming MCR syntheses of pyrazoles. The review will primarily focus on two major categories: two-carbon and three-carbon building blocks as key intermediates, while other special cases will be summarized separately.

## Review

### (3 + 2)-Cyclocondensation – C_3_-building blocks as key intermediates

The majority of the numerous pyrazole syntheses known in the literature are based on the condensation of 1,3-dielectrophiles and their synthesis equivalents as C_3_-building blocks, along with hydrazines as N_2_-building blocks [25–38]. Conceptually, the en route generation of these C_3_-building blocks or their transformation with additional components besides hydrazines is a logical entry to MCR syntheses of pyrazoles. Therefore, 1,3-dielectrophiles encompass 1,3-dicarbonyl compounds and α,β-unsaturated carbonyl compounds, including alkenoyl and alkynoyl systems, across various oxidation states of the carbonyl groups and their derivatives.

#### 1,3-Dicarbonyl compounds as key intermediates

The most common and classic synthesis of ring-forming pyrazoles is the cyclocondensation of 1,3-dicarbonyl compounds with hydrazines (Knorr synthesis) [42–43]. Therefore, the in situ generation of 1,3-dicarbonyl compounds and their one-pot transformation pave the way for MCR syntheses of pyrazoles.

**Scheme 1:**
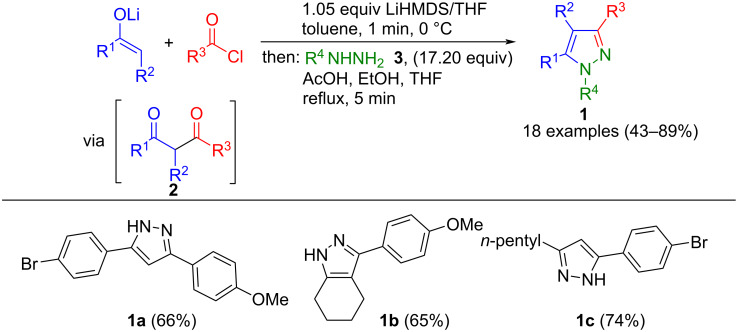
Consecutive three-component synthesis of pyrazoles **1** via in situ-formed 1,3-diketones **2** [44].

1,3-Dicarbonyl compounds can, for example, be generated in situ from enolates and carboxylic acid chlorides. They can be converted to the corresponding pyrazoles **1** in a consecutive multicomponent reaction with hydrazines **3** (Scheme 1) [44]. It is important for a chemoselective synthesis that the 1,3-diketones **2** formed are not further acylated, which is prevented by using LiHMDS as a base. The method provides good to excellent yields and tolerates diverse functional groups. However, one limitation of the synthesis is that the reaction with methylhydrazine leads to two different regioisomers.

**Scheme 2:**
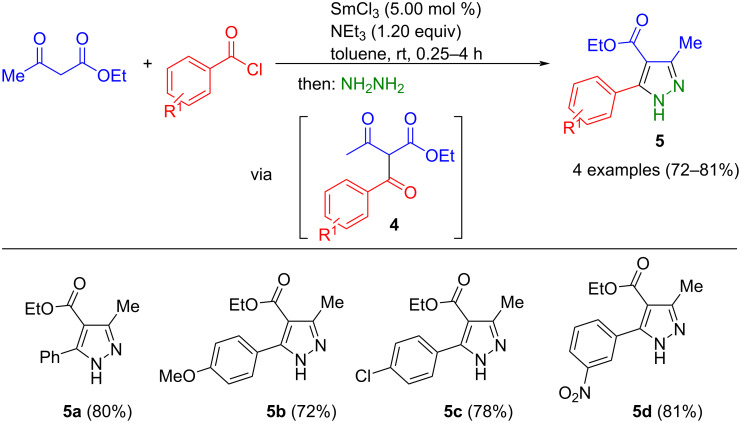
Consecutive three-component synthesis of 4-ethoxycarbonylpyrazoles **5** via SmCl_3_-catalyzed acylation of ethyl acetoacetate [45].

Shen et al. used a concept developed by them for the C-acylation of β-ketoesters for the one-pot synthesis of pyrazoles. SmCl_3_-catalyzed acylation of these yields the 1,3-diketones **4**, and after cyclization with hydrazine, 3,4,5-substituted pyrazoles **5** are formed (Scheme 2) [45]. The Lewis acid catalyst accelerates the reaction via participation in the formation of β-diketonate complexes.

**Scheme 3:**
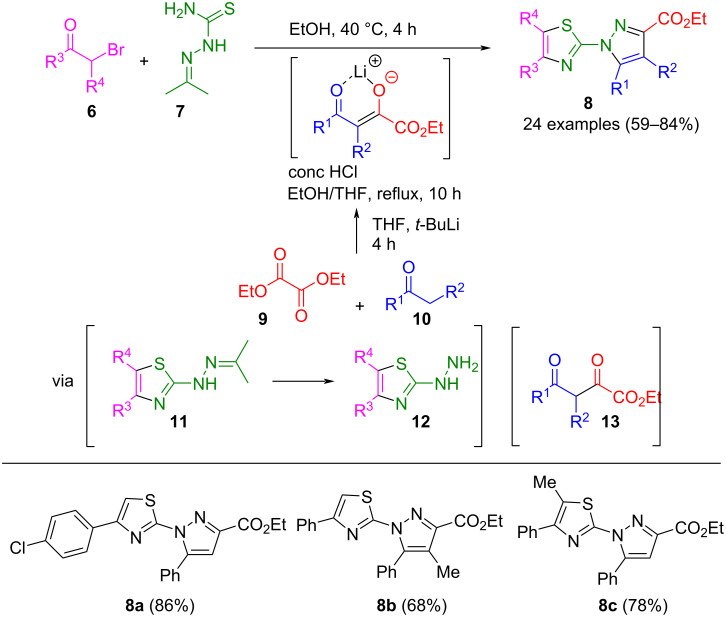
Consecutive four-component synthesis of 1-(thiazol-2-yl)pyrazole-3-carboxylates **8** [51].

Other carbonyl compounds suitable for pyrazole synthesis are 2,4-diketoesters **13**. These intermediates can be prepared from diethyl oxalate (**9**) and alkylphenones **10** through a sterically hindered Claisen condensation, producing a six-membered lithium enolate salt. Subsequent cyclocondensation with hydrazines concludes the formation of pyrazoles. However, this process could not be performed as a one-pot synthesis, as the solvent had to be exchanged after the Claisen step [46–50]. Gu et al. succeeded in adapting this concept for the one-pot synthesis of 1-(thiazol-2-yl)pyrazole-3-carboxylates **7** (Scheme 3) [51]. Starting from β-bromocarbonyl compounds **6** and 2-(propane-2-ylidene)thiosemicarbazide (**7**) thiazolylhydrazones **11** are formed via Hantzsch's thiazole synthesis. After acidic deprotection to thiazolylhydrazines **12**, these react with enolates of 2,4-diketoesters, which are intermediaries prepared in a separate reaction vessel, yielding the corresponding (thiazol-2-yl)pyrazoles **8**. However, hydrazines are not tolerated in this consecutive four-component reaction, as the 1,3,4-thiadiazine synthesis competes with the thiazole synthesis.

**Scheme 4:**
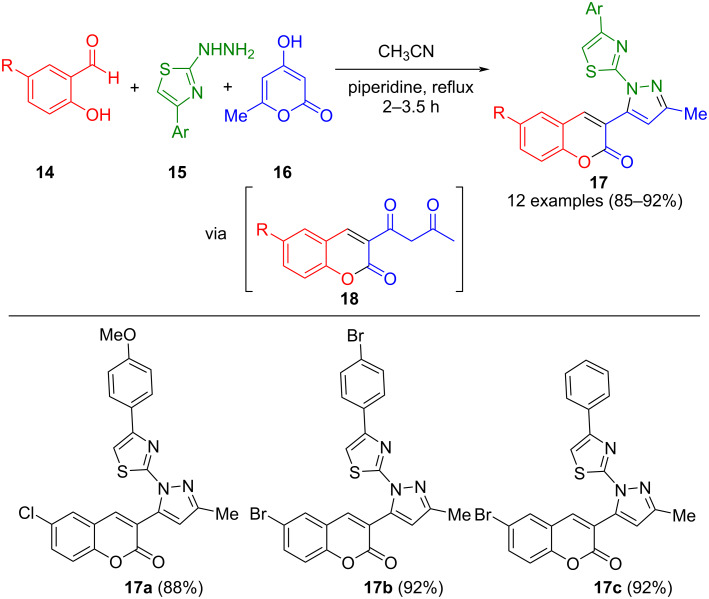
Three-component synthesis of thiazolylpyrazoles **17** via in situ formation of acetoacetylcoumarins **18** [52].

Salicylaldehydes **14** and 4-hydroxy-6-methyl-2*H*-pyran-2-one (**16**) can also be used to produce 1,3-dicarbonyl compounds **18** by Knoevenagel condensation and subsequent cyclization. This approach was also used to synthesize pyrazoles since the intermediary-formed diketone **18** forms the corresponding pyrazoles **17** in a Knorr reaction with 2-hydrazinyl-4-phenylthiazoles **15** in a one-pot process (Scheme 4) [52]. Piperidine was used as a catalyst for the Knoevenagel condensation. Remarkably, product **17a** shows cytotoxic activity against Hep G2 hepatocellular carcinoma and MCF-7 breast carcinoma cell lines.

**Scheme 5:**
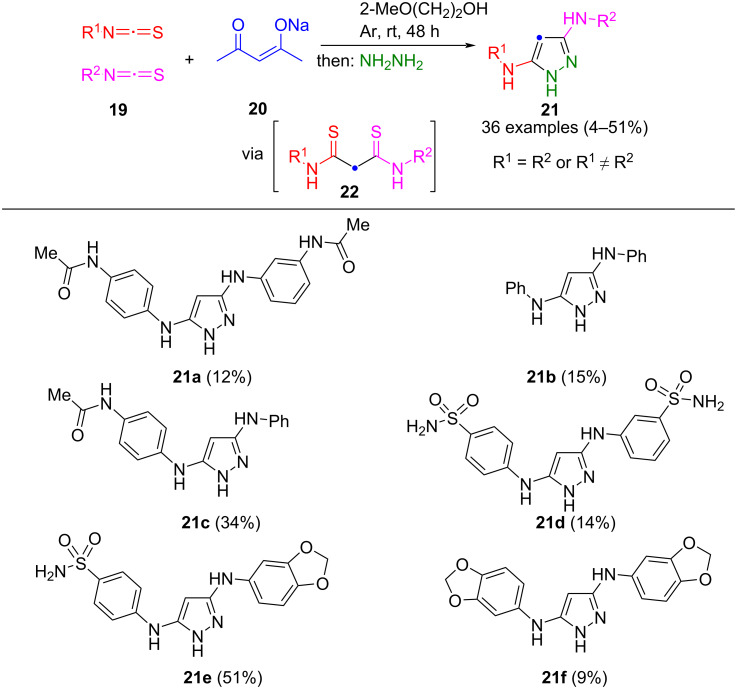
Consecutive pseudo-four-component and four-component synthesis of pyrazoles **21** from sodium acetylacetonate (**20**), isothiocyanates **19**, and hydrazine [53].

The synthesis of 3,5-bis(arylamino)pyrazoles **21** involves the preparation of bisarylthioamides **22** via nucleophilic addition and double retro-Claisen condensation of isothiocyanates **19** and acetylacetonate **20**. Extension of the sequence by cyclization with hydrazine leads to the pyrazole products **21** (Scheme 5) [53]. The method works best with acetylacetonates, since deprotonation of other diketones with NaOEt leads to deactivation of the isothiocyanates. A limitation of the method is the formation of three different regioisomers when employing different isothiocyanates **19**. However, easy separation by LC–MS could be achieved, as demonstrated for one-pot process generated pyrazoles **21a**–**f**. The reaction demonstrates high tolerance to steric hindrance and electronic factors, enabling the synthesis of various 3,5-bis(arylamino)pyrazoles **21**.

**Scheme 6:**
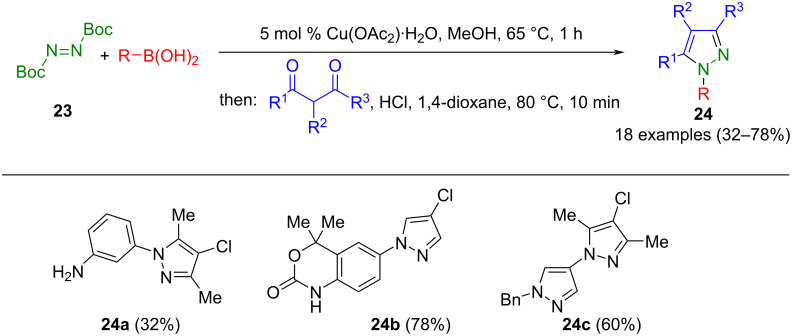
Consecutive three-component synthesis of 1-substituted pyrazoles **24** from boronic acids, di(Boc)diimide **23**, and 1,3-dicarbonyl compounds [54].

In multicomponent syntheses, arylhydrazines were also presented in situ and could be cyclized with 1,3-dicarbonyl compounds in a one-pot process. This process yields *N*-functionalized pyrazoles, expanding the scope of available compounds. A common challenge in pyrazole synthesis is the synthetic accessibility of hydrazines. To circumvent this limitation, arylboronic acids can be coupled with Boc-protected diimide **23** under copper catalysis to form the hydrazine precursor in situ. Subsequent removal of the Boc groups and cyclocondensation with 1,3-dicarbonyl compounds leads to the formation of pyrazoles **24** in a one-pot process (Scheme 6) [54]. This versatile method enables the introduction of various functional groups at position 1 of the pyrazole ring. In addition, it can also be employed in synthesizing celecoxib, an active pharmaceutical ingredient.

**Scheme 7:**
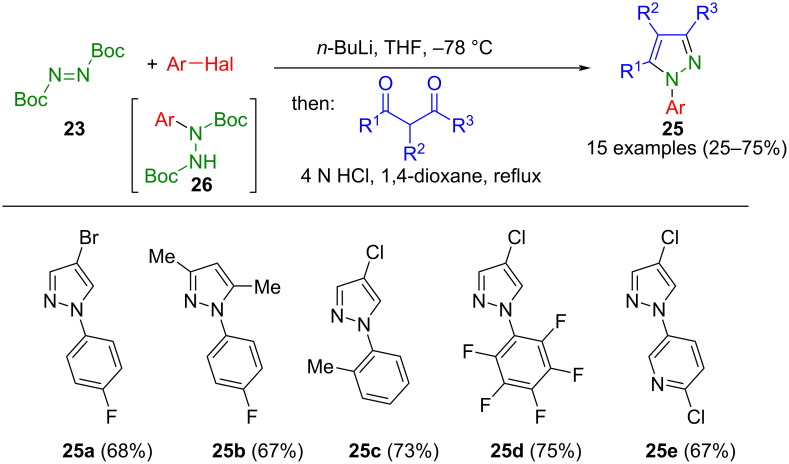
Consecutive three-component synthesis of *N*-arylpyrazoles **25** via in situ formation of aryl-di(Boc)hydrazines **26** [56].

Starting from di-*tert*-butyldiazocarboxylate (**23**), aryl-substituted di-Boc-hydrazines **26** were prepared by the addition of aryllithium species generated in situ by lithium-halogen exchange of aryl halides [55]. Gerstenberger et al. used this entry for the one-pot synthesis of *N*-arylpyrazoles **25,** as depicted in Scheme 7 [56].

In a consecutive process, after removing the protecting groups through acidic cleavage and cyclocondensation with 1,3-dicarbonyl compounds, the corresponding pyrazoles **25** were formed in a one-pot procedure. Interestingly, both β-aminoacrolein and β-aminovinylketones can also serve as substrates according to this method. However, attempts to carry out the reaction sequence with unprotected hydrazine were unsuccessful. Alternatively, Mitsunobu reagent **23** can be reacted with in situ generated benzyne (from *o*-(trimethylsilyl)phenyl triflate) to provide the hydrazides for the concomitant cyclocondensation with 1,3-dicarbonyl compounds [57].

**Scheme 8:**
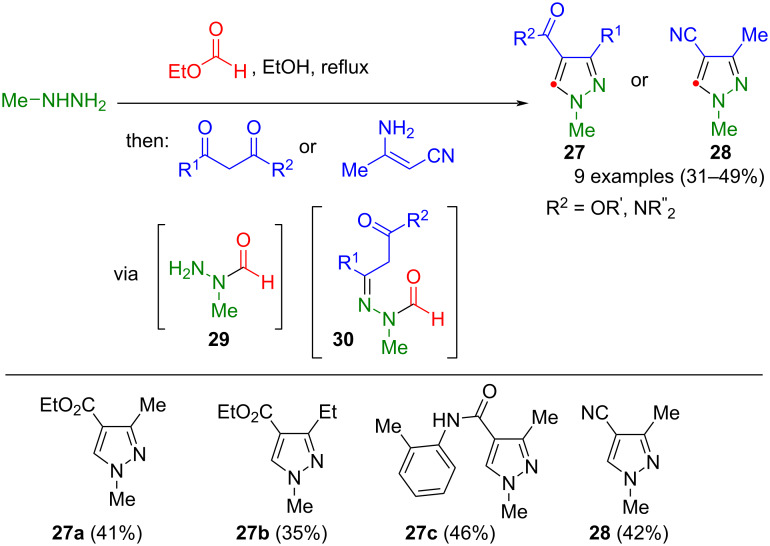
Consecutive three-component synthesis of 1,3,4-substituted pyrazoles **27** and **28** from methylhydrazine, ethyl formate, and β-ketoesters, β-ketoamides or 3-aminocrotonitrile [58].

In their program to synthesize regioselectively substituted pyrazoles, Raw and Turner established the one-pot preparation of triply substituted pyrazoles **27** and **28** via 1-formyl-1-methylhydrazine (**29**), generated in situ by reacting methylhydrazine and ethyl formate (Scheme 8) [58]. Upon reaction with β-ketoesters, hydrazone **30** is formed, which reacts via intramolecular Knoevenagel condensation to give the corresponding pyrazoles **27**. The method tolerates β-ketoesters with alkyl substituents and various ketoamides. In addition, an example could be synthesized starting from 3-aminocrotononitrile to yield pyrazole **28**. The authors explicitly mention that aryl ketones do not transform using this method.

**Scheme 9:**
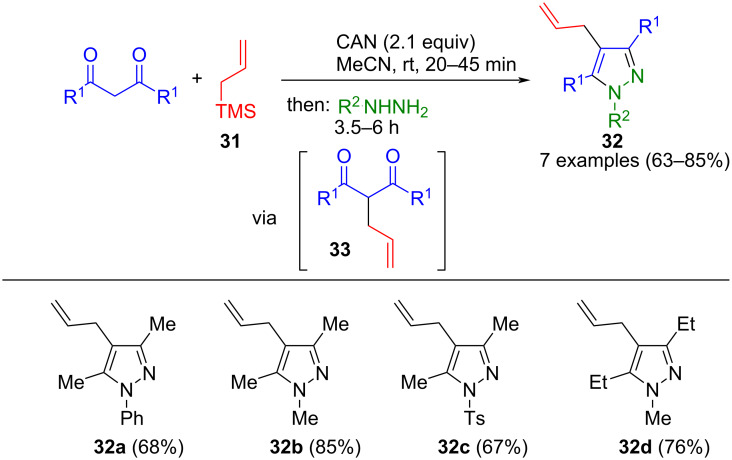
Consecutive three-component synthesis of 4-allylpyrazoles **32** via oxidative allylation of 1,3-dicarbonyl compounds [59].

Besides, the functionalization of 1,3-dicarbonyl compounds and their subsequent conversion into pyrazoles can be conducted in a one-pot fashion. For instance, oxidative allylation of 1,3-dicarbonyl compounds using allyltrimethylsilane (**31**) in the presence of ammonium cerium(IV) nitrate (CAN) provides access to allylated 1,3-dicarbonyl compounds **33** that are transformed with hydrazines to the corresponding pyrazoles **32** in a one-pot process (Scheme 9) [59]. CAN serves a dual role as a Lewis acid and an oxidizing agent. However, a significant limitation of the method is that it only tolerates aliphatic 1,3-dicarbonyl compounds, as the reaction with aromatic carbonyl compounds leads to very low yields. Improved yields for the latter can be achieved by isolating the functionalized 1,3-dicarbonyl compound **33** after the first step. Instead of allyl, a cyano group can also be introduced at position 2 of the 1,3-dicarbonyl compound using TsCN [60]. Cyclization of the intermediates leads to the corresponding 4-cyanopyrazoles.

**Scheme 10:**
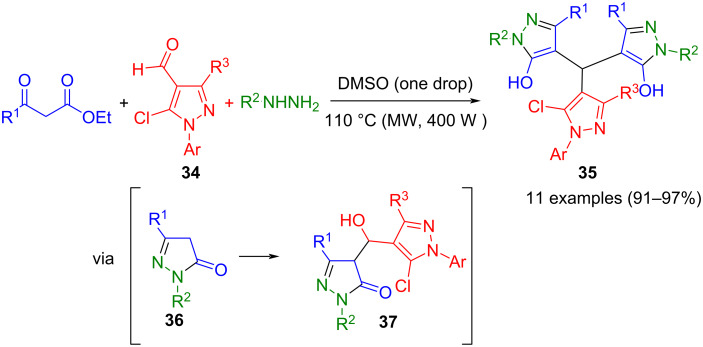
Pseudo-five-component synthesis of tris(pyrazolyl)methanes **35** [61].

Beyrati and Hasaninejad presented a microwave-assisted pseudo-five-component synthesis of tris(pyrazolyl)methanes **35**, where first β-ketoesters and hydrazines form pyrazolones **36**. One equivalent reacts in a Knoevenagel condensation with 4-formylpyrazole **34** to give pyrazolidine pyrazole **37**, while the second equivalent undergoes a Michael addition to form the corresponding trispyrazole **35** (Scheme 10) [61]. Similarly, aromatic aldehydes furnish the corresponding bispyrazolylmethanes either under ultrasound irradiation in low-boiling solvents [62] or under Ag/TiO_2_ catalysis [63].

**Scheme 11:**
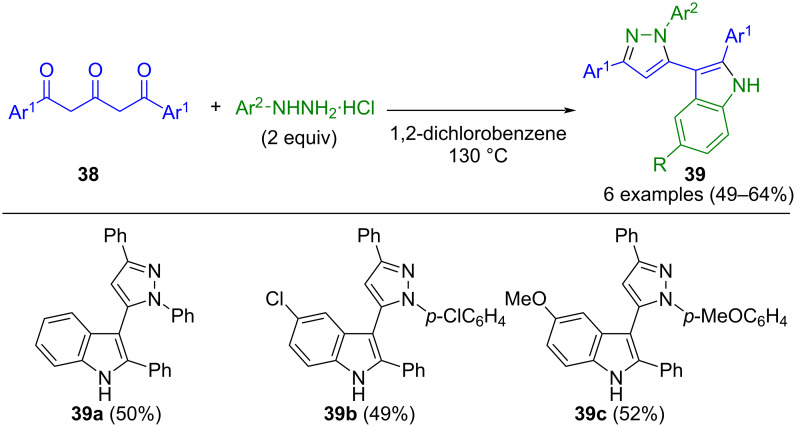
Pseudo-three-component synthesis of 5-(indol-3-yl)pyrazoles **39** from 1,3,5-triketones **38** [64].

In addition to 1,3-dicarbonyl compounds, 1,3,5-tricarbonyl compounds **38** are also interesting building blocks in pyrazole synthesis. Through the reaction of 1,5-diaryl-1,3,5-pentanetriones **38** with hydrazines, Knorr synthesis of pyrazoles and Fischer indole synthesis can be combined in a pseudo-three-component fashion to give 5-(indol-3-yl)pyrazoles **39** (Scheme 11) [64]. The reaction proceeds in moderate to good yields and can be conducted either consecutively or as a domino reaction. However, one limitation of the method is that the pyrazole formation is not regiospecific and leads to two isomers.

**Scheme 12:**
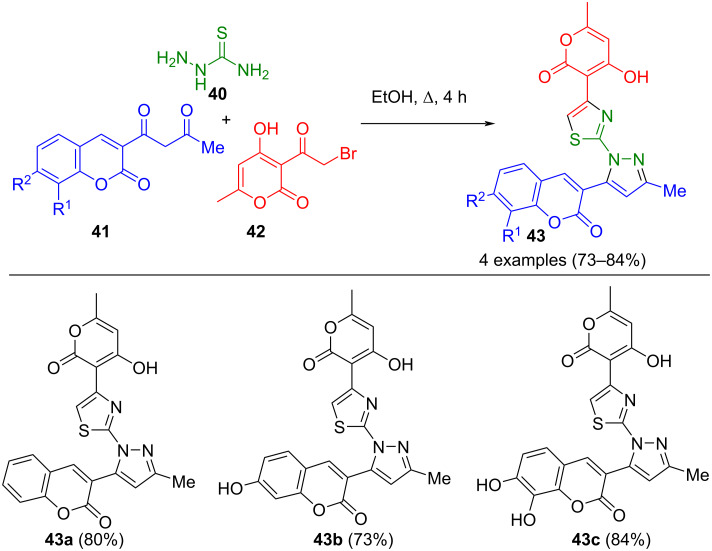
Three-component synthesis of thiazolylpyrazoles **43** [65].

Functionalized hydrazines are versatile building blocks in organic synthesis. For instance, hydrazinecarbothioamide (**40**) can be used to synthesize bisheterocycles. Mohamed et al. were able to combine Hantzsch thiazole and Knorr pyrazole synthesis with this building block. Thiazolyl-pyrazolyl-chromenes **43** were synthesized in good yields from substituted 3-acetoacetylcoumarin **41**, 3-bromoacylpyran **42**, and semicarbazide **40** (Scheme 12) [65]. Alternatively, the corresponding chromenes can replace the 3-bromoacylpyrans. A notable advantage of this process is its catalyst-free nature and the achievement of good regioselectivity. Due to the high heterocycle density, this method holds promise for the development of biologically active substances. In addition to acetoacetylcoumarins, the synthesis was also successfully conducted using acetylacetone [66].

**Scheme 13:**
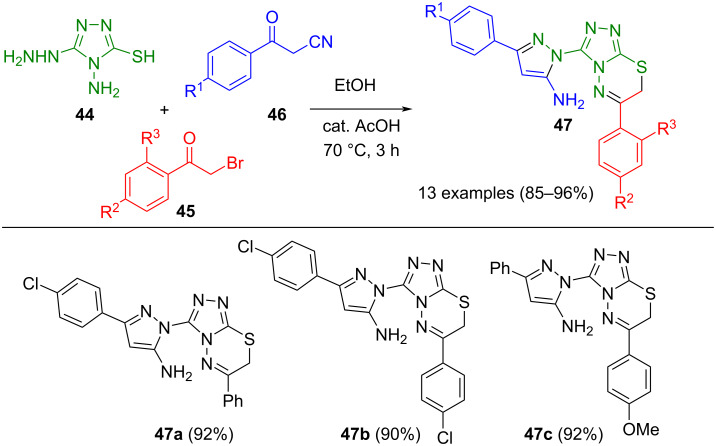
Three-component synthesis of triazolo[3,4-*b*]-1,3,4-thiadiazin-3-yl substituted 5-aminopyrazoles **47** [67].

Likewise, complex functionalized pyrazoles, such as triazolo[3,4-*b*]-1,3,4-thiadiazin-3-yl substituted 5-aminopyrazoles **47**, can be accessed from polyfunctional hydrazine derivatives via multicomponent reactions. For the preparation of pyrazoles **47**, 4-amino-5-hydrazinyl-4*H*-1,2,4-triazole-3-thiol (**44**), phenylacyl bromides **45**, and benzoylacetonitriles **46** were chosen as starting materials (Scheme 13) [67]. Thereby, benzoylacetonitrile and the hydrazinyl moiety constitute the pyrazole nucleus, while phenylacyl bromide condenses with the thiol and amino groups to form the thiadiazinyl moiety. In addition to bromoacetylchromenone, phenylacetyl bromide can also be used as an alternative starting material.

**Scheme 14:**
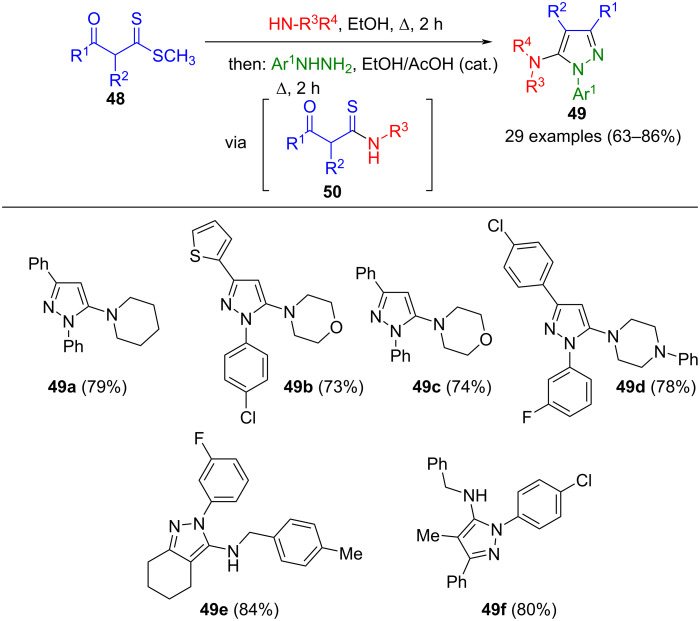
Consecutive three-component synthesis of 5-aminopyrazoles **49** via formation of β-oxothioamides **50** [68].

1,3-Dielectrophilic β-oxodithioesters **48** react with primary or cyclic aliphatic amines, resulting in the formation of β-oxothioamides **50**, which can then be directly converted into the corresponding 5-aminopyrazoles **49** with aromatic hydrazines in the presence of catalytic amounts of acetic acid (Scheme 14) [68]. The process exhibits regioselectivity, with 3,4-fused pyrazoles being accessible when cyclic β-oxodithioesters are employed as substrates. However, aromatic amines cannot be successfully employed in the sequence.

#### Alkenoyl derivatives as key intermediates

The Michael addition and cyclocondensation of hydrazines and α,β-unsaturated carbonyl compounds represent a standard approach for synthesizing pyrazolines, which readily oxidize to aromatic pyrazoles in the presence of ambient atmosphere. On the other hand, if good leaving groups are bound to the hydrazine, such as in tosylhydrazine, elimination directly yields aromatic pyrazoles. In cases where the α,β-unsaturated carbonyl compounds contain a heteroatom in the β-position, aromatization is triggered by elimination under redox-neutral conditions.

**Scheme 15:**
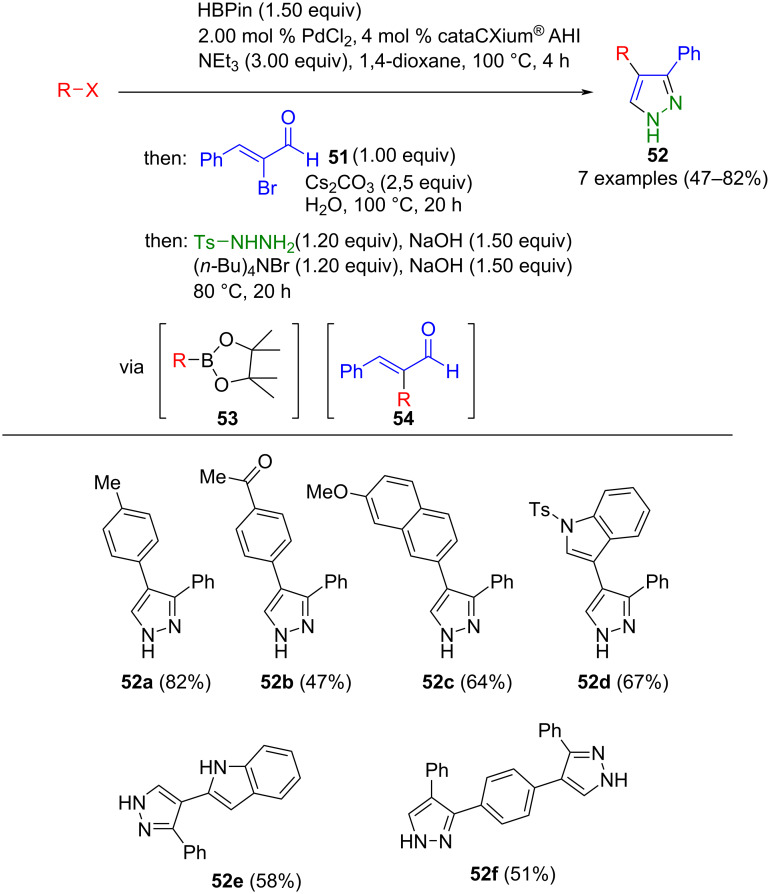
Synthesis of 3,4-biarylpyrazoles **52** from aryl halides, α-bromocinnamaldehyde, and tosylhydrazine via MBSC/cyclocondensation-elimination sequence [70].

Tasch et al. successfully coupled aryl halides with α-bromocinnamaldehyde (**51**) using a Masuda borylation Suzuki cross-coupling (MBSC) [69] approach without reducing the reactivity of the Michael system. In this one-pot procedure, the borylation of aryl halides with pinacolborane gives aryl pinacolyl boronates **53**, which are then coupled with bromoenal **51** to generate the intermediary enal **54**. Subsequent cyclization with tosylhydrazine and elimination to give the corresponding pyrazoles **52** (Scheme 15) [70]. The cross-coupling introduced various aryl substituents at position 4. Furthermore, using 1,4-diiodobenzene as a starting material allows access to bispyrazole derivatives. According to Beller [71], using bisadamantyl-type phosphane ligands is crucial for the selectivity in this reaction.

**Scheme 16:**
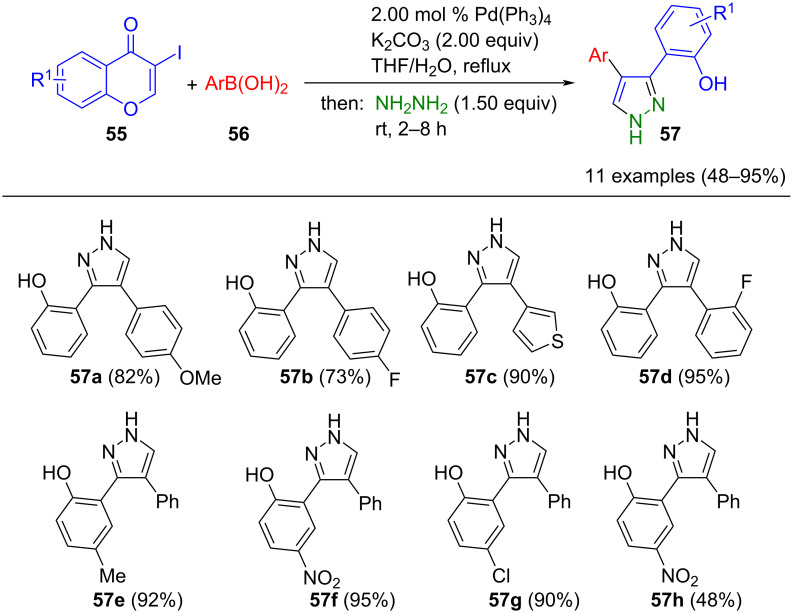
Consecutive three-component synthesis of 3,4-substituted pyrazoles **57** from iodochromones **55** by Suzuki coupling and subsequent ring opening-ring closing cyclocondensation with hydrazine [72].

Suzuki coupling can also serve for the functionalization of iodochromones **55**, which, as α,β-unsaturated ketones, undergo ring opening under the reaction conditions, followed by Michael addition–cyclocondensation. Xie et al. devised a method to synthesize 3,4-substituted pyrazoles **57** from iodochromones **55**, arylboronic acids **56**, and hydrazines (Scheme 16) [72]. During the Suzuki step, electronically and sterically diverse substituents were successfully coupled to chromones. Notably, a single regioisomer is formed when methylhydrazine is employed, likely due to the more electron-rich internal nitrogen atom reacting with the Michael system. When phenylhydrazine is used, an inverse reactivity is observed [73–75]. Remarkably, when hydroxyethylhydrazine is utilized, a mixture of regioisomers is formed, likely attributed to steric hindrances.

**Scheme 17:**
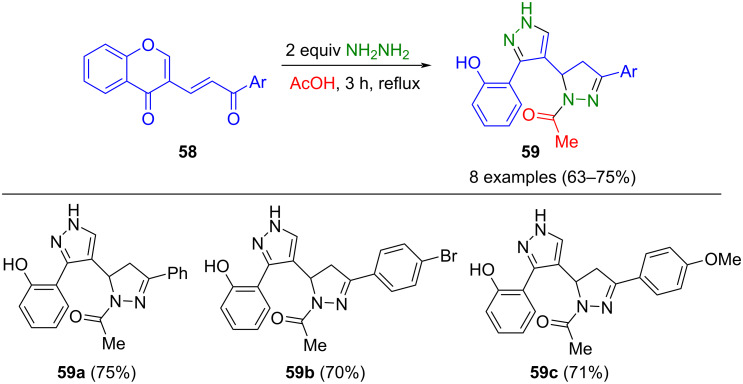
Pseudo-four-component synthesis of pyrazolyl-2-pyrazolines **59** by ring opening/ring closing cyclocondensation with hydrazine [76].

α,β-Unsaturated ketones embedded and tethered in chromene systems **58** were successfully employed in a pseudo-five-component reaction with hydrazine in boiling acetic acid to give the corresponding 4-acylpyrazolinylpyrazoles **59** (Scheme 17) [76]. The Michael addition–cyclocondensation of the α,β-unsaturated ketone with hydrazine and acetic acid forms a 1-acylpyrazoline, while the chromene moiety and hydrazine form the pyrazole nucleus by ring opening/ring closing cyclocondensation. Upon oxidation with DDQ, the pyrazolylpyrazoline products can be readily converted into the corresponding bispyrazoles.

**Scheme 18:**
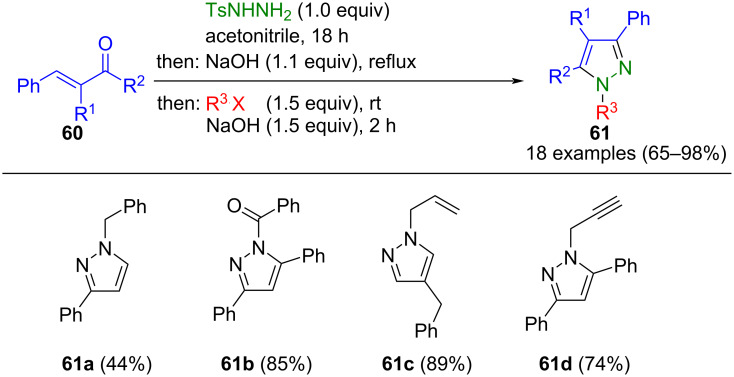
Consecutive three-component synthesis of pyrazoles **61** [77].

The α,β-unsaturated carbonyl compounds **60** can undergo cyclization with tosylhydrazine in situ to form pyrazoles **61** under alkaline conditions, with the tosyl group acting as a leaving group. Upon deprotonation at position 1 by a base, followed by nucleophilic substitution of halides, *N*-functionalized pyrazoles **61** are accessible in a consecutive three-component fashion (Scheme 18) [77]. The reaction exhibits regioselectivity in many cases, attributed to steric influences.

**Scheme 19:**
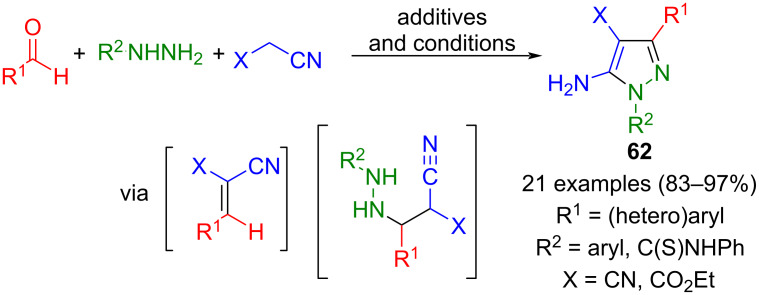
Three-component synthesis of pyrazoles **62** from malononitrile, aldehydes, and hydrazines [78–90].

Aldehydes and nitriles with an activated methylene group, such as malononitrile, are well-known for being potent precursors of α,β-unsaturated cyano derivatives, which in turn can cyclocondense with hydrazines to furnish aminopyrazoles after oxidation. Hasanijedad and Firoozi developed a one-pot process for synthesizing 5-aminopyrazoles **62** in a three-component fashion (Scheme 19) [78]. Interestingly, hydrazine acts both as a Brønsted base in the Knoevenagel reaction and as a component for ring formation. Cyclization is completely regioselective when arylhydrazines are used; with methylhydrazine, the ratio is greater than 10:1. In addition to malononitrile, derivatives with reduced nucleophilicity (X = CO_2_R) are also tolerated in the method. Starting from dialdehydes, bridged pyrazoles are accessible. However, one limitation of this strategy is the inability to use aliphatic aldehydes. Various modifications of the transformation using these substrates have been reported, where catalysts and/or conditions are varied. The one-pot process can be conducted in various solvents such as in PEG 400 [79], ionic liquids like 1-butyl-3-methylimidazolium hydroxide ((Bim)OH) [80], the nanoionic liquid 1-methylimidazolium trinitrocarbide ([[HMIM]C(NO_2_)_3_]) [81], *N*-methylpyridinium tosylate (NMPYT) [82], or glucose-based strong eutectic solvents (DES) [83]. Catalysis can also be achieved using molecular iodine [84], AlCl_3_ [85], sodium ascorbate [86], and even solid-state and nanoparticle-mediated catalysts like CuO/ZrO_2_ [87], Fe_3_O_4_@Si@MoO_2_ [88], caspacin-cyclodextrin functionalized magnetite nanoparticles (CPS CD) [89], and Mg-Fe hydrolactite catalysts (C-Mg-Al HAT-3) [90].

**Scheme 20:**
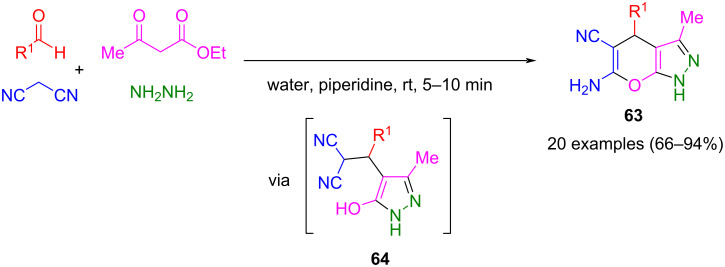
Four-component synthesis of pyrano[2,3-*c*]pyrazoles **63** [91].

With β-ketoesters, the method can be extended to a four-component synthesis. Initially, β-ketoesters react with hydrazine to form pyrazolones, while a Knoevenagel reaction between malononitrile and aldehyde simultaneously generates a Michael system. Both intermediates undergo cyclization following a Michael addition to yield the corresponding pyrano[2,3-*c*]pyrazoles **63** (Scheme 20) [91]. Safaei-Ghomi et al. succeeded in isolating the intermediately formed pyrazole **64** [92]. Since pyrano[2,3-*c*]pyrazoles are fused heterocycles of interest, catalytic methods for their synthesis have been extensively reviewed [93].

**Scheme 21:**
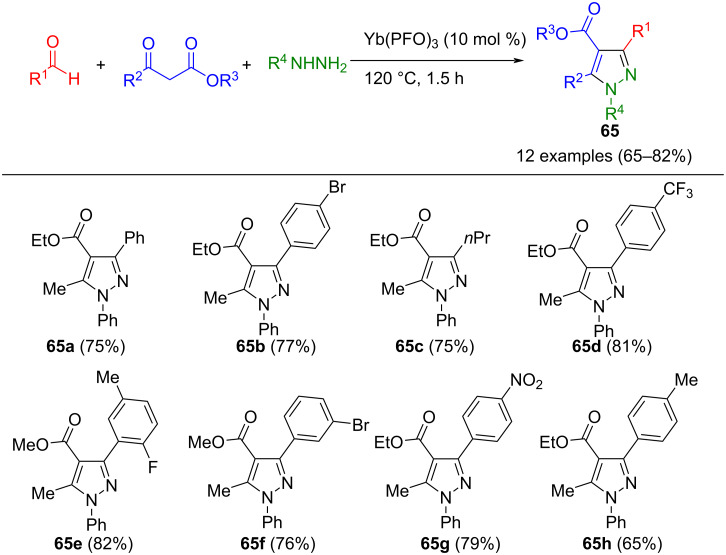
Three-component synthesis of persubstituted pyrazoles **65** from aldehydes, β-ketoesters, and hydrazines [95].

When hydrazones undergo cyclization with β-ketoesters or ketones, the stability of the enol form of the carbonyl compounds plays a crucial role in the reaction. Recognizing this, a series of multicomponent reactions has been developed to synthesize pyrazole-4-carboxylates. Shen et al. used Yb(PFO)_3_ (PFO: perfluorooctanoate), a mild and highly efficient catalyst shown to be effective in the Mannich reaction [94], to synthesize these pyrazoles **65** (Scheme 21) [95].

The Lewis acid catalyst activates and stabilizes the enol tautomer of β-ketoesters, facilitating their cyclization with intermediary formed hydrazones to yield 5-hydroxypyrazolines. After subsequent interaction with atmospheric oxygen, the product **65** is formed. This method exhibits a high degree of substituent tolerance, and aliphatic compounds generally lead to higher yields. A special feature of the method is the regioselectivity with asymmetric diketones. In addition to β-ketoesters, acetylacetone can be used in the concept. The same research group employed ethyl 4,4,4-trifluoroacetoacetate as a substrate in this method. The pyrazoline oxidation proved to be critical, and the addition of IBX ensured the completion of the reaction sequence [96]. A notable advantage of this method is the ability to reuse the catalyst multiple times. In addition to Yb(PFO)_3_, zinc triflate can also serve as an effective catalyst for the synthesis of pyrazoles **65**. It also catalyzes the oxidation of pyrazoline, which leads to increased yields. In addition to fully substituted pyrazoles, bispyrazoles can also be synthesized [97]. Other Lewis acid catalysts employed in this method include chloride-functionalized silica gel (SiO_2_Cl) [98] and Sc(OTf)_3_ [99]. Importantly, all reactions of this method are conducted without solvent.

**Scheme 22:**
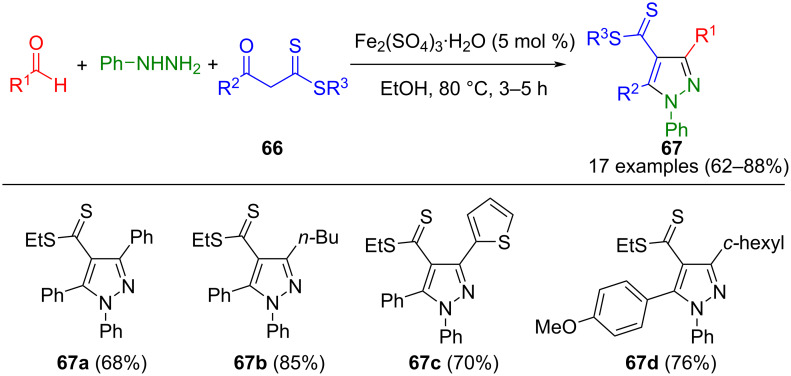
Three-component synthesis of pyrazol-4-carbodithioates **67** [100].

In a similar approach, Khan et al. succeeded in synthesizing pyrazole-4-carbodithioates **67**. The products are prepared from phenylhydrazine, aldehydes, and alkyl-3-oxo-3-arylpropane dithioates **66** catalyzed by iron sulfate (Scheme 22) [100]. In this method, aliphatic aldehydes as substrates also increase yields.

**Scheme 23:**
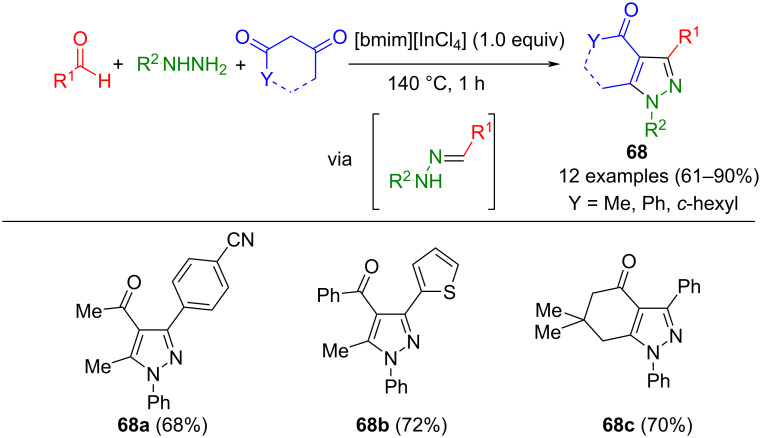
Regioselective three-component synthesis of persubstituted pyrazoles **68** catalyzed by ionic liquid [bmim][InCl_4_] [101].

The ionic liquid [bmim][InCl_4_] can be used as a catalyst for the one-pot synthesis of pyrazoles **68** from 1,3-diketones, aldehydes, and hydrazines (Scheme 23) [101]. The synergistic effect between anion and cation favors high regioselectivity, and high yields can be observed in the process for both electron-rich and electron-poor aldehydes. In addition to fully substituted pyrazoles, the method also provides access to anellated products.

**Scheme 24:**
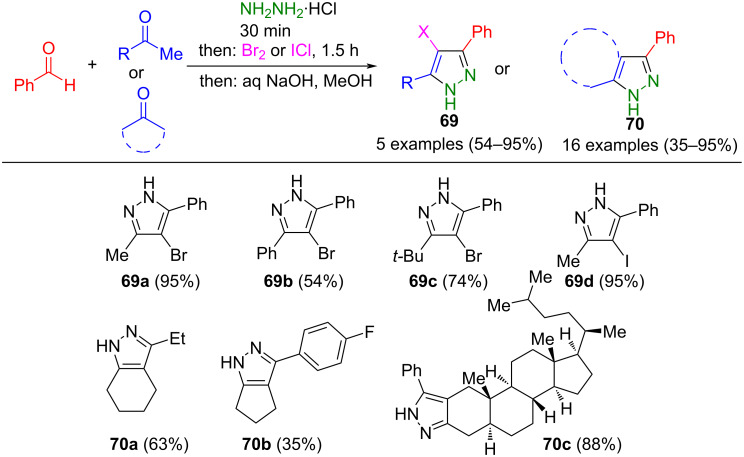
Consecutive three-component synthesis of 4-halopyrazoles **69** and anellated pyrazoles **70** [102].

Lellek's team has developed an alternative method for cyclizing hydrazones. In contrast to the previously mentioned method, intermediately generated hydrazones were cyclized with simple ketones to pyrazolines. The oxidation to the corresponding 4-halo-substituted pyrazoles **69** can be achieved in a one-pot fashion by halogenation with iodine chloride or elemental bromine (Scheme 24) [102]. When cyclic ketones are used, fused products **70** are formed. Notably, this process is compatible with both aromatic and aliphatic aldehydes.

**Scheme 25:**
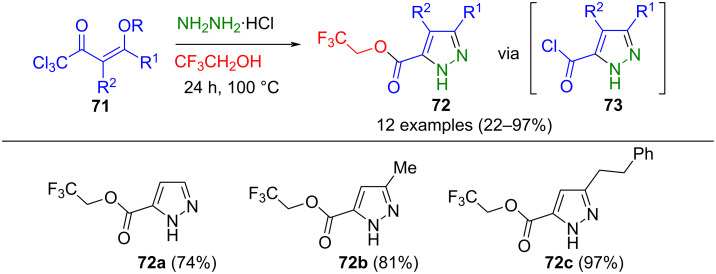
Three-component synthesis of 2,2,2-trifluoroethyl pyrazole-5-carboxylates **72** [103].

Pyrazoles with fluorinated functionalities were synthesized via a three-component reaction utilizing trifluoroethanol both as a solvent and reactant. Gonçalves et al. developed a method whereby 1,1,1-trichloro-6-phenyl-2,4-hexanedione was generated in situ through acid hydrolysis of 1,1,1-trichloro-4-methoxy-6-phenyl-3-hexen-2-ones **71**. Subsequent cyclization with hydrazine hydrochloride followed by hydrolysis of the trichloromethyl group led to **73**. This intermediate was then reacted with 2,2,2-trifluoroethanol (TFE) as a solvent, yielding 2,2,2-trifluoroethyl pyrazole-5-carboxylates **72** (Scheme 25) [103]. A limitation of the method is that methanol formed in the first reaction step also reacts with the acid chloride to produce unwanted byproducts.

**Scheme 26:**
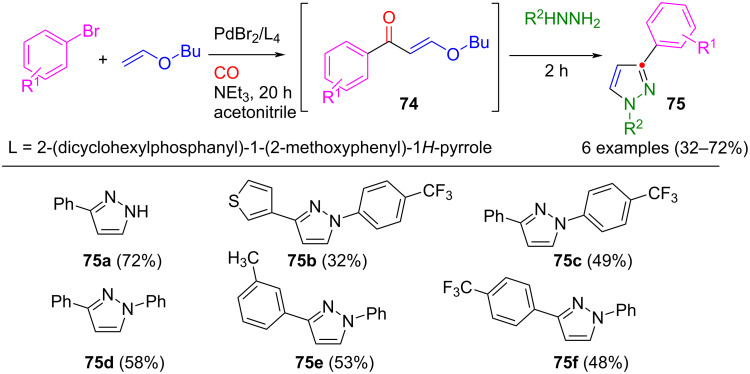
Synthesis of pyrazoles **75** in a one-pot process via carbonylative Heck coupling and subsequent cyclization with hydrazines [104].

The carbonylative Heck coupling of aryl bromides with butyl vinyl ether enables the synthesis of 3-alkoxyalkenones **74**, presenting an elegant route. Subsequently, these compounds undergo a three-component reaction with various hydrazines, forming 1,3-substituted pyrazoles **75** (Scheme 26) [104]. However, one limitation is the formation of 1,5-substituted pyrazoles as byproducts in proportions of less than 10%.

**Scheme 27:**
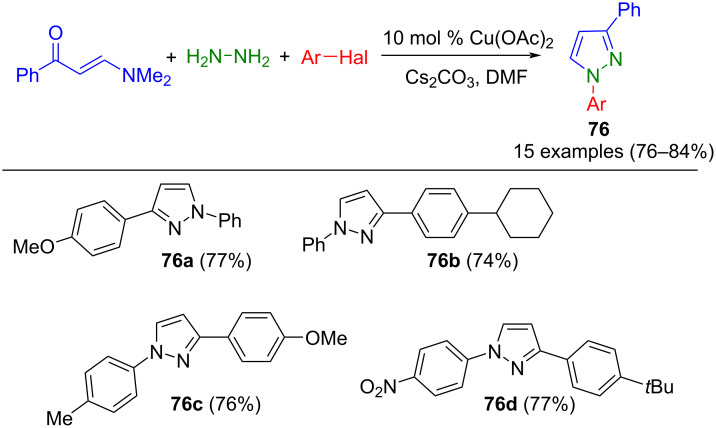
Copper-catalyzed three-component synthesis of 1,3-substituted pyrazoles **76** [105].

Copper catalysis opens the unique opportunity to form C–N bonds, e.g., for N-functionalization of pyrazoles in a one-pot fashion. Raghunadh et al. developed a process for the synthesis of 1,3-substituted pyrazoles **76**, introducing aryl substituents at position 1. This protocol involves the utilization of enaminones, hydrazine, and various aryl halides as substrates, resulting in the formation of 1,3-substituted pyrazoles **76** (Scheme 27) [105].

Mechanistic studies have indicated that in this domino reaction, the initial formation of 3-substituted pyrazoles occurs through the cyclization of the hydrazine with the enaminone, followed by a Ullmann coupling with aryl halides to form the corresponding 1,3-substituted pyrazoles **76**. The method tolerates both sterically demanding and electronically versatile aryl moieties.

**Scheme 28:**
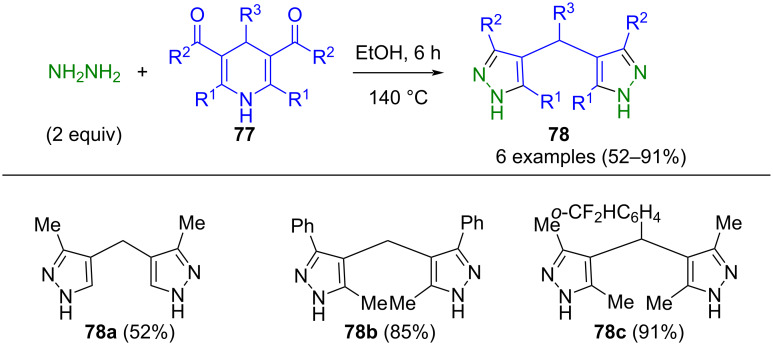
Pseudo-three-component synthesis of bis(pyrazolyl)methanes **78** by ring opening-ring closing cyclocondensation with hydrazine [106].

Enaminones embedded in dihydropyridines, specifically 3,5-acyl-1,4 dihydropyridines **77**, are well suited for undergoing ring opening-ring closing cyclocondensation with hydrazine in a pseudo-multicomponent reaction to give bis(pyrazolyl)methanes **78** (Scheme 28) [106].

Interestingly, the use of ester derivatives leads to the formation of the corresponding pyrazolone derivatives, thereby providing access to both bis(pyrazolyl) and bis(pyrazolonyl)methanes.

**Scheme 29:**
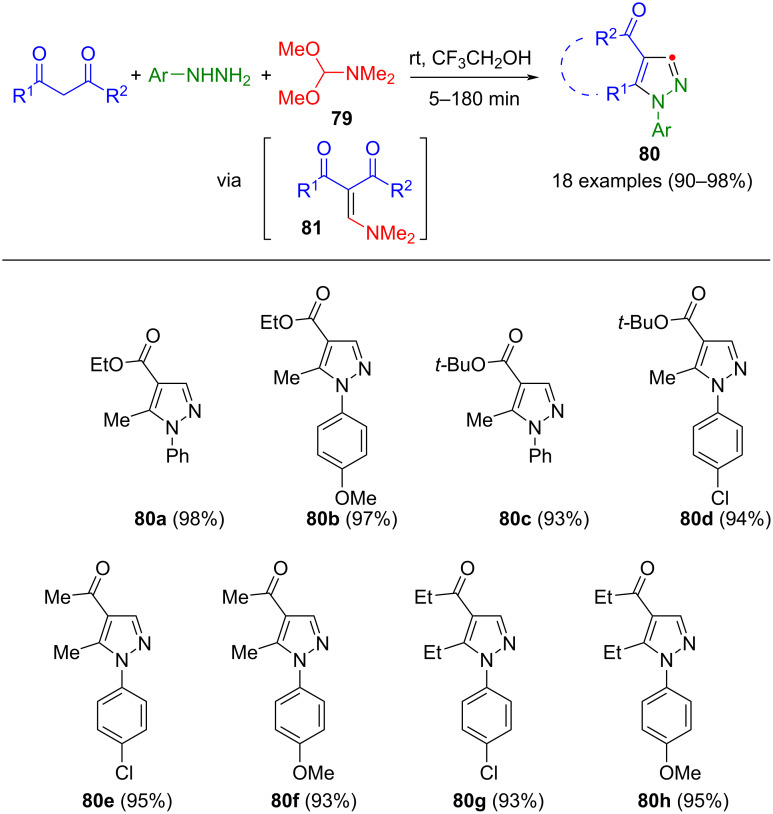
Three-component synthesis of 1,4,5-substituted pyrazoles **80** [107].

Enaminones **81** can be generated as intermediates by condensation of 1,3-dicarbonyl compounds and DMF-dimethylacetal (DMFDMA, **79**). The reaction is catalyzed by the solvent 2,2,2-trifluoroethanol, which coordinates with the carbonyl groups, and thereby stabilizing the enol form of the 1,3-dicarbonyl compounds. In the presence of hydrazines, cyclization furnishes the corresponding 1,4,5-substituted pyrazoles **80** in a one-pot process (Scheme 29) [107]. It is noteworthy that for arylhydrazines, all components can be initially present in the reaction vessel, whereas methylhydrazine is added dropwise to the enaminone to prevent the formation of regioisomers. The versatility of this method is highlighted by its tolerance towards both β-ketoesters and aliphatic 1,3-dicarbonyl compounds. Moreover, it can be performed under solvent-free conditions [108] and is applicable to the synthesis of anellated pyrazoles [109]. In addition, arylsulfonate-substituted pyrazoles can be accessed using this protocol [110].

**Scheme 30:**
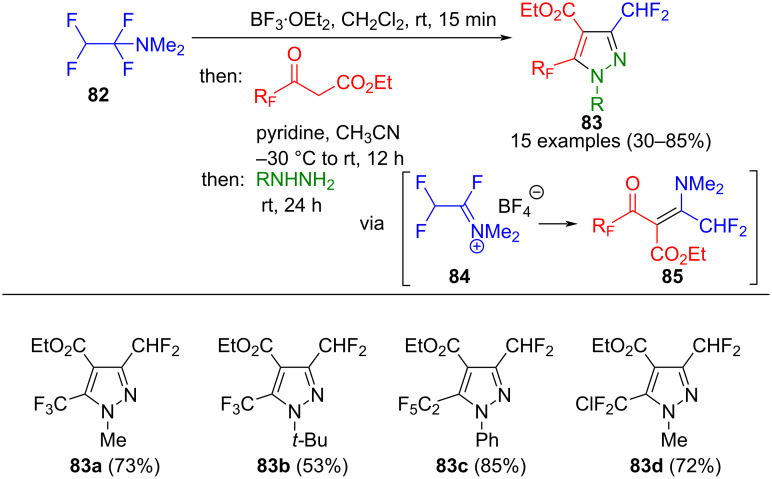
Consecutive three-component synthesis of 3,5-bis(fluoroalkyl)pyrazoles **83** [111].

Due to their significance in pharmaceutical chemistry [11], the formation of fluoropyrazoles from fluorinated 1,3-dielectrophiles represents a crucial synthetic pathway. For example, 1,1,2,2-tetrafluoro-*N*,*N*-dimethylethan-1-amine (TFEDMA) (**82**) can be activated using BF_3_·OEt_2_ to generate an iminium salt **84** with increased electrophilicity. Subsequently, this intermediate reacts with various β-ketoesters to form enaminones **85**. The consecutive three-component reaction culminates in cyclization with substituted hydrazines, leading to the regioselective formation of 5-fluoroalkylpyrazoles **83** (Scheme 30) [111].

Following subsequent saponification, pyrazole-4-carboxylic acids can be obtained. The process tolerates various fluorinated β-ketoesters. Moreover, both aliphatic and aromatic hydrazines can be used, with methyl and phenylhydrazines leading to increased yields. The one-pot synthesis of pyrazoles **83** can be carried out on a 100 g scale [112], and, in addition to β-ketoesters, enamino esters are tolerated in the method [113].

**Scheme 31:**
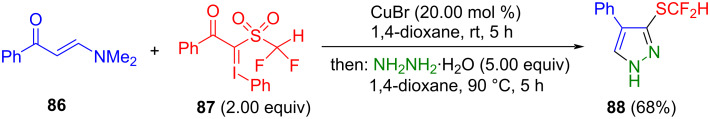
Consecutive three-component synthesis of difluoromethanesulfonyl-functionalized pyrazole **88** [114].

Starting from enaminone **86** functionalization, the hypervalent iodine compound **87** facilitates the introduction of a difluoromethanesulfonyl group in the copper(I) bromide-mediated consecutive three-component synthesis of difluoromethanesulfonyl-functionalized pyrazole **88** (Scheme 31) [114]. In addition to pyrazoles, functionalized pyrimidines can also be prepared in a one-pot process using this method.

**Scheme 32:**
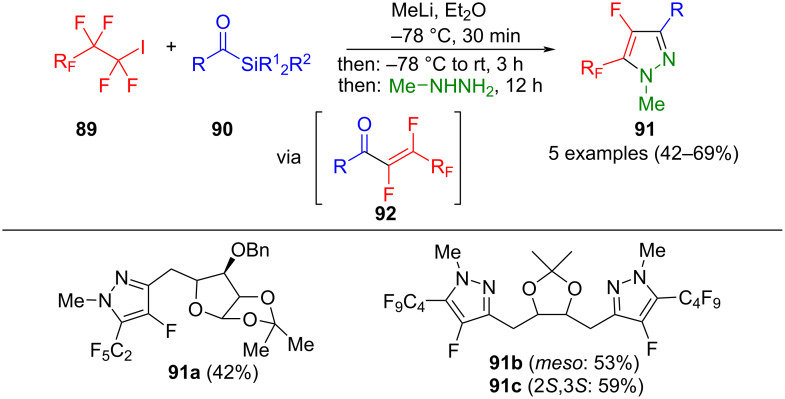
Consecutive three-component synthesis of perfluoroalkyl-substituted fluoropyrazoles **91** [115].

In 2001, Bouillon et al. reported a one-pot process for synthesizing fluorinated pyrazoles. Initially, a lithium–halogen exchange was carried out on fluorinated iodoalkanes **89**. Subsequent reaction with acylsilanes **90** leads to alcohols, which react by Brook rearrangement to enoxysilanes and form the intermediate **92**. Following a Michael addition/cyclocondensation with methylhydrazine, 4-fluoropyrazoles **91** are regioselectively formed (Scheme 32) [115]. Bouillon hypothesized that the Michael addition occurs via the most nucleophilic nitrogen atom of the methylhydrazine at the β-fluorine atom of intermediate **92**. An excess of hydrazine is used in the method, as hydrofluoric acid is formed during the reaction.

**Scheme 33:**
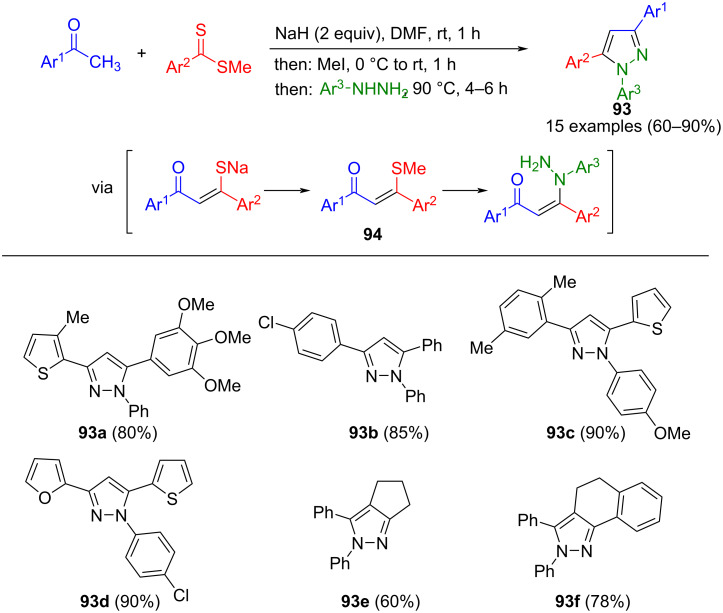
Regioselective consecutive three-component synthesis of 1,3,5-substituted pyrazoles **93** [116].

Derivatives of α,β-unsaturated ketones, specifically 1,3-bis(hetero)aryl-3-(methylthio)-2-propenones **94**, are accessible through Claisen-type thioacylation of methylketones, followed by methylation. These intermediates serve as precursors for the concomitant cyclization with arylhydrazines in a consecutive multicomponent reaction to regioselectively give pyrazoles **93** (Scheme 33) [116]. The presence of two equivalents of sodium hydride deprotonates the acidic α-NH of arylhydrazine, thereby determining the regioselective attack in addition–elimination sequence furnishing a hydrazine enaminone intermediate, and ultimately leading to pyrazole **93** after cyclization. An advantage of this method for preparing 1,3,5-substituted pyrazoles is its tolerance towards a wide range of substituents.

**Scheme 34:**
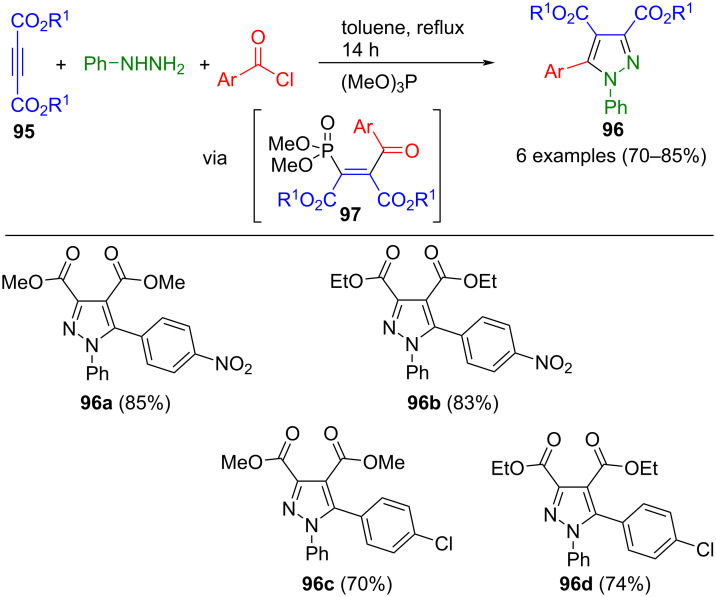
Three-component synthesis of pyrazoles **96** mediated by trimethyl phosphite [117].

Trimethyl phosphite can be added to acetylene dicarboxylates **95** to generate a zwitterion that readily reacts with electrophiles. This zwitterion undergoes a rearrangement similar to a Michaelis–Arbuzov reaction, yielding an α,β-unsaturated ketone **97**. The domino sequence concludes by cyclocondensation of intermediate **97** with phenylhydrazine, ultimately affording fully substituted pyrazoles **96** after the elimination of dimethyl phosphonate (Scheme 34) [117].

**Scheme 35:**
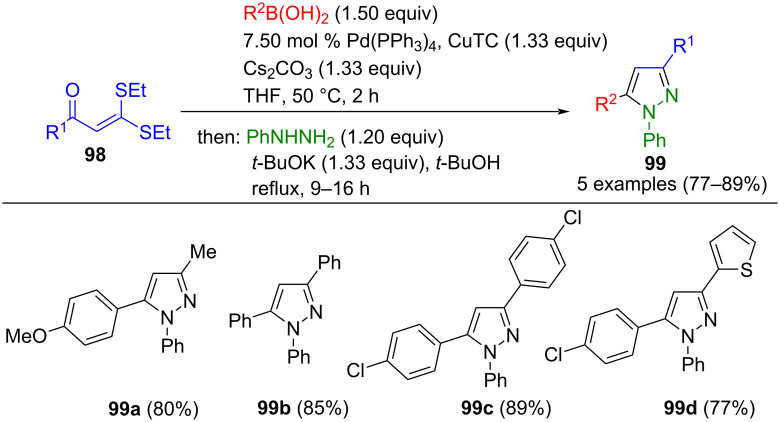
One-pot synthesis of pyrazoles **99** via Liebeskind–Srogl cross-coupling/cyclocondensation [118].

β-Thioalkyl-α,β-unsaturated ketones **98** are S,S-ketene acetals capable of undergoing Liebeskind–Srogl coupling with boronic acids and phenylhydrazine in a consecutive three-component reaction to give pyrazoles **99** (Scheme 35) [118].

**Scheme 36:**
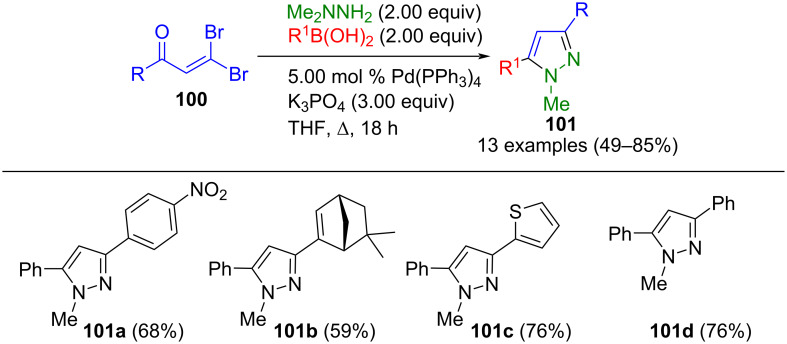
Synthesis of 1,3,5-substituted pyrazoles **101** via domino condensation/Suzuki–Miyaura cross-coupling of β,β-dibromenones **100** and 1,1-dimethylhydrazine [119].

Beltrán-Rodil et al. used a similar strategy to synthesize 1,3,5-substituted pyrazoles **101** by Suzuki coupling of β,β-dibromenones **100**, boronic acids, and 1,1-dimethylhydrazine in a three-component reaction (Scheme 36) [119]. The intermediary 5-bromopyrazoles formed by condensation/*N*-demethylation/cyclization sequence subsequently react by Suzuki–Miyaura cross-coupling in a one-pot fashion. The reaction is regioselective; however, methylhydrazine cannot be employed as a substrate.

#### Alkynoyl derivatives as key intermediates

Alkynones and alkynoyl derivatives also constitute a class of α,β-unsaturated carbonyl compounds, yet in a different oxidation state. Therefore, Michael addition and cyclocondensation with hydrazines proceed directly towards pyrazoles without oxidation. The formation of pyrazoles from alkynals and alkynones as three-carbon building blocks by cyclocondensation with hydrazine has been known for quite some time [120]. Recent advancements have sparked inquiries into whether three-carbon alkynoyl building blocks [121–122] could be generated catalytically [123], thereby facilitating reactions under mild reaction conditions and opening novel one-pot pathways for consecutive multicomponent syntheses of pyrazoles.

**Scheme 37:**
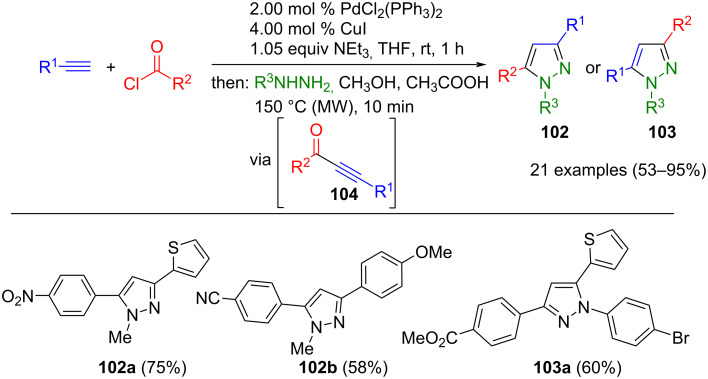
Consecutive three-component synthesis of 1,3,5-trisubstituted pyrazoles **102** and **103** by Sonogashira alkynylation–cyclocondensation sequences from alkynes, (hetero)aroyl chlorides, and hydrazines [127].

Sonogashira alkynylation of terminal alkynes and (hetero)aroyl chlorides furnishes alkynones **104** under mild reaction conditions [124–125]. Without isolation alkynones **104**, hydrazines react directly by Michael addition–cyclocondensation, affording 1,3,5-trisubstituted pyrazoles **102** and **103** with remarkable regioselectivity (Scheme 37) [126–127].

The nucleophilicity of the attacking hydrazine nitrogen atom is essentially controlled by the regioselectivity. In the case of aliphatic hydrazines, the secondary nitrogen atom exhibits a higher electron density, thereby dictating the preferred formation of pyrazoles **102**. Conversely, for arylhydrazines, resonance stabilization of the secondary nitrogen atom attenuates the nucleophilicity on the secondary nitrogen, furnishing regioisomeric product **103**. This latter inverse regioselectivity is typical for arylhydrazines, and due to a lower overall nucleophilicity, often forcing conditions for the cyclocondensation are necessary [73].

Most advantageously, this methodology tolerates many polar functional groups and allows access to pyrazole libraries from simple starting materials (alkynes, acid chlorides, hydrazines) in good to excellent yield. Notably, pyrazoles **102** and **103** demonstrate intense luminescence both in solution and in the solid state [127]. Furthermore, even sugar-functionalized pyrazoles have been accessed by this approach [128], and it was readily implemented in a continuous flow reactor [129]. Besides traditional Sonogashira catalyst systems, highly reactive and reusable immobilized Pd-complexes, such as [MCM-41-2N-Pd(OAc)_2_] [130] and Pd-SH-silica bound catalysts [131] have also been successfully employed in this three-component pyrazole synthesis. Interestingly, the latter catalyst system does not require a copper cocatalyst.

**Scheme 38:**
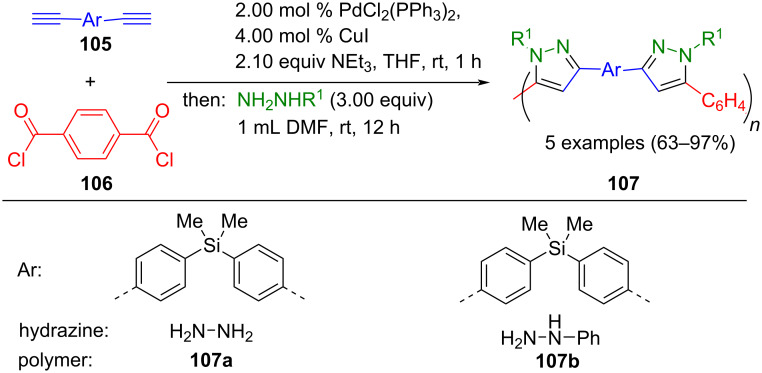
Polymer analogous consecutive three-component synthesis of pyrazole-based polymers **107** [132].

Tang's research group further extended polymer synthesis by using the consecutive three-component approach in a polymer analogous fashion with diynes **105**, terephthaloyl chloride (**106**), and hydrazines to synthesize high molecular weight pyrazole-based polymers **107** (Scheme 38) [132]. The materials **107** fluoresce in THF solutions, form thin films, and possess high thermal stability and high optical refraction.

**Scheme 39:**
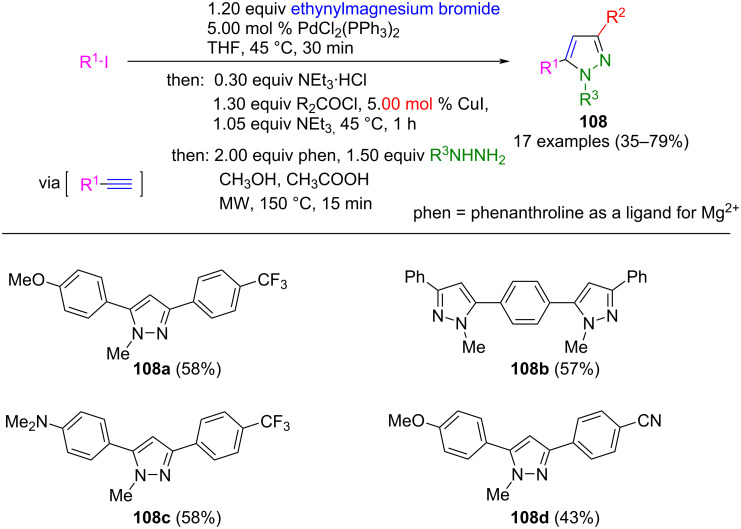
Synthesis of 1,3,5-substituted pyrazoles **108** by sequentially Pd-catalyzed Kumada–Sonogashira cyclocondensation sequence [136].

Functionalized alkynes can alternatively be prepared in situ by a Kumada coupling [133] of aryl iodides and ethynylmagnesium bromide [134]. The Pd catalyst is reused in the subsequent Sonogashira coupling for the synthesis of alkynones in the sense of sequential catalysis [135]. Following this, a one-pot process involving Michael addition/cyclocondensation with hydrazine derivatives leads to the corresponding pyrazoles. Since magnesium ions are formed during the Kumada coupling, the additive phenanthroline must be added during the cyclization step to prevent the coordination of the magnesium ions and subsequent hydrazine inactivation. The diversity-oriented nature of this consecutive four-component synthesis was used to synthesize 17 different donor/acceptor-substituted pyrazoles **108** in moderate to good yields (Scheme 39) [136]. The synthesis of bispyrazoles was also possible in a pseudo-seven-component reaction starting from 1,4-diiodobenzene. The product **108c** showed strong solvatochromism.

**Scheme 40:**
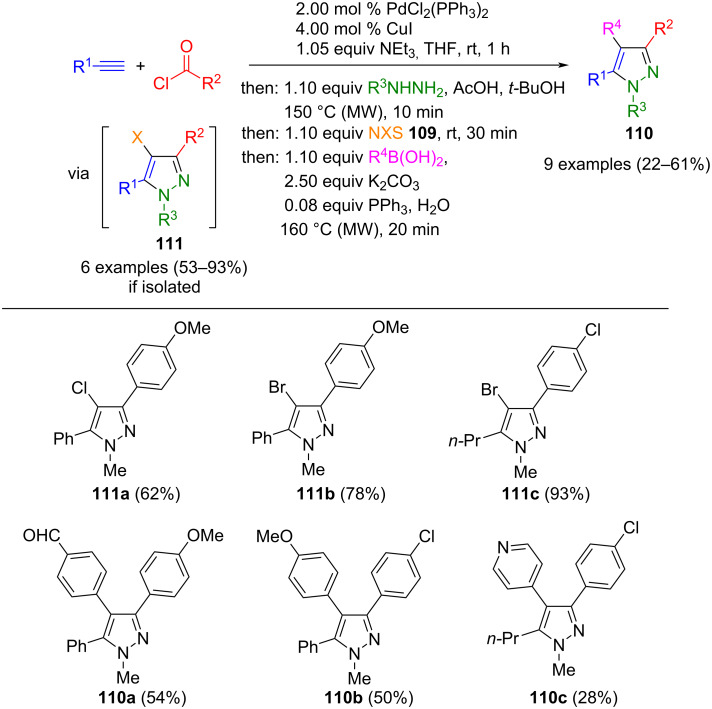
Consecutive four-step one-pot synthesis of 1,3,4,5-substituted pyrazoles **110** [137].

The synthesis of pyrazoles from terminal alkynes, acyl chlorides, and hydrazines [124,126] can be extended by subsequent halogenation at position 4 with *N*-halosuccinimide **109** (X = Cl, Br). The resulting 4-halopyrazoles **111** can either be isolated or undergo further Suzuki coupling with arylboronic acids. Both couplings use the Pd catalyst in a sequential fashion. To increase the yields, both cyclization and Suzuki coupling are carried out with microwave support. Some of the synthesized 4 halopyrazoles **111** and their Suzuki products **110** fluoresce blue in solution and have quantum yields of 29–72 % (Scheme 40) [137].

**Scheme 41:**
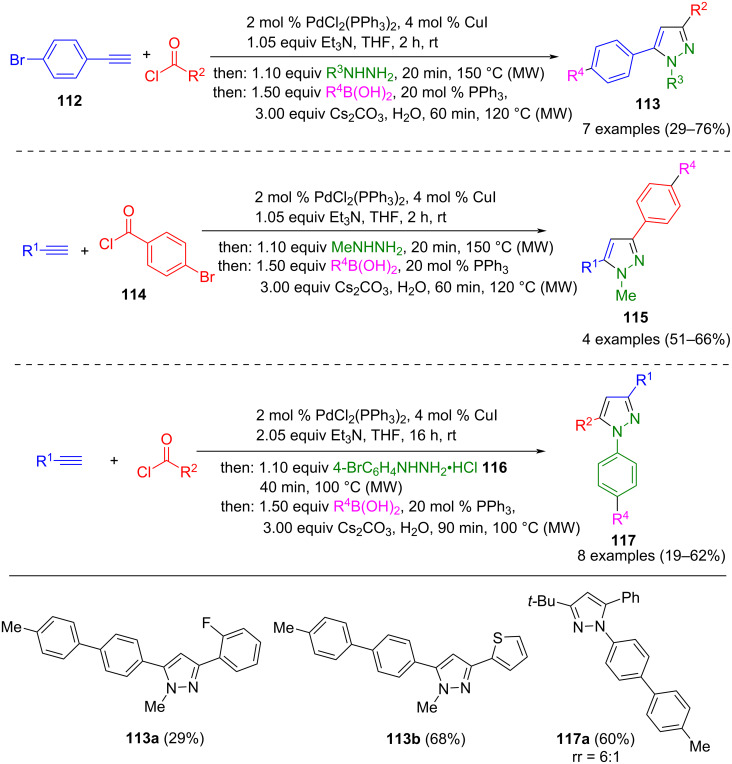
Four-component synthesis of pyrazoles **113**, **115**, and **117** via Sonogashira coupling and subsequent Suzuki coupling [138].

The Suzuki coupling can also be used for the functionalization of pyrazoles. For this purpose, *p*-bromo-substituted terminal alkynes **112**, acyl chlorides **114**, and arylhydrazines **116** are used as starting materials. The *p*-bromophenylpyrazoles presented in situ can be reacted with boronic acids or boronic acid esters in a sequentially Pd-catalyzed coupling towards biaryl-substituted pyrazoles **113**, **115**, and **117** (Scheme 41) [138]. Furthermore, a pseudo-five-component reaction pathway enables the synthesis of 3,5-bis(biphenyl)-1-methyl pyrazole. To stabilize the catalyst, additional triphenylphosphane is added as a ligand during the Suzuki coupling. The resulting products fluoresce blue, with the five biaryl-substituted derivatives **113** showing the highest quantum yields of up to 97%.

**Scheme 42:**
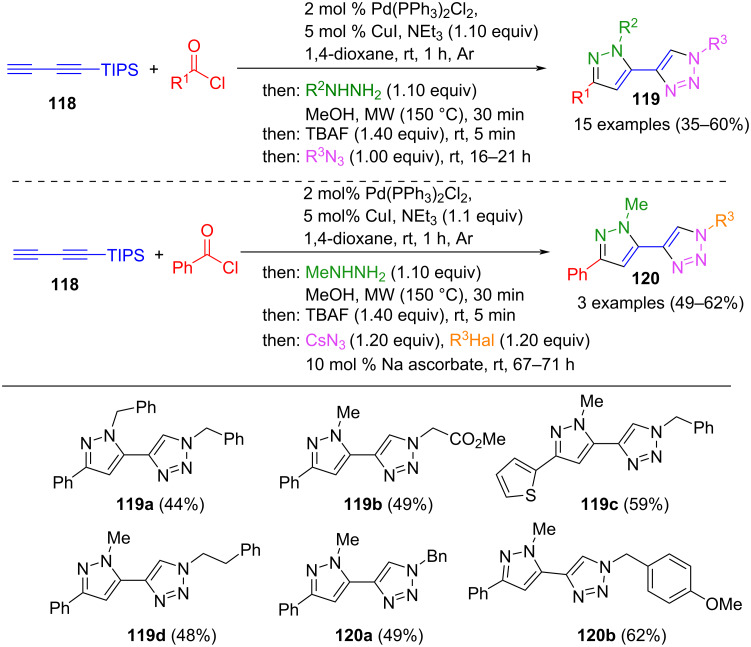
Consecutive four- or five-component synthesis for the preparation of 4-pyrazoly-1,2,3-triazoles **119** and **120** [139].

The Sonogashira coupling can also be effectively integrated with the CuAAC (copper-catalyzed azide–alkyne cycloaddition) reaction, offering a powerful tool for synthesizing diverse molecular architectures. In a consecutive multicomponent reaction, pyrazoles were first presented in a Sonogashira cyclization sequence from (triisopropylsilyl)butadiyne (**118**). Subsequent immediate desilylation and Click reaction with organoazides lead to 4-pyrazolyl-1,2,3-triazoles **119** (Scheme 42) [139]. Notably, in some examples, it was even possible to synthesize the organoazides in situ from alkyl halides and cesium azide for the synthesis of compounds **120**. The choice of the hydrazine substituent represents a limitation, as no aromatic substituents are tolerated in the strategy due to the reduced reactivity. However, due to the building blocks’ simplicity, various 4-pyrazolyl-1,2,3-triazoles are accessible.

**Scheme 43:**
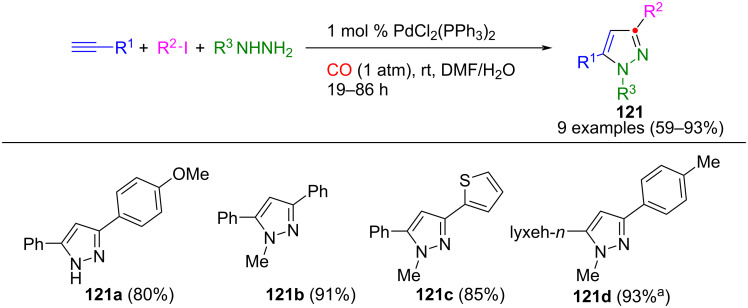
Four-component synthesis of pyrazoles **121** via alkynone formation by carbonylative Pd-catalyzed coupling [140]. ^a^5.00 mol % PdCl_2_(PPh_3_)_2_, 2.00 mol % CuI.

Aryl iodides undergo conversion to the corresponding alkynones in aqueous ammonia through a palladium-catalyzed carbonylative coupling with terminal alkynes under 1 atm of carbon monoxide. Building upon this discovery, these intermediates were cyclized with hydrazines to give pyrazoles **121** in a one-pot process (Scheme 43) [140]. This alkynone generation fulfills two functions: on the one hand, it acts as an activating reagent in the coupling reaction and, on the other, as a ring-forming component. Notably, this process can only be carried out as a domino reaction. For the coupling of aliphatic alkynes, copper iodide is necessitated as a co-catalyst [141]. In addition to classic Sonogashira catalysts, phosphane-free palladium [Pd(NN)] chelate complexes can also be used, which promote excellent regioselectivity [142]. A variation of the reaction is possible with Mo(CO)_6_ as a carbon monoxide source. This allows the use of phenylhydrazine, which is not tolerated under standard conditions. However, the synthesis of unsymmetric pyrazoles produces two regioisomers [143].

Recently, Tu et al. reported a similar method for the regioselective synthesis of trisubstituted pyrazoles via palladium-catalyzed oxidative carbonylative Sonogashira alkynylation and cyclocondensation with arylhydrazines [144].

**Scheme 44:**
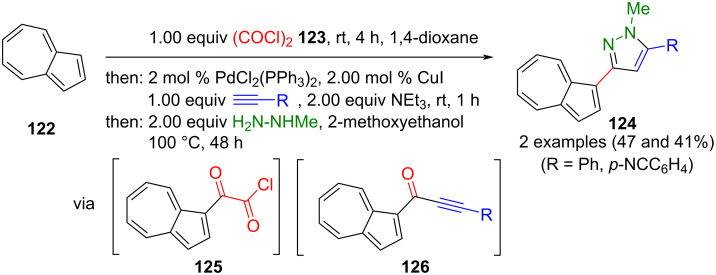
Preparation of 3-azulenyl pyrazoles **124** by glyoxylation, decarbonylative Sonogashira coupling, and subsequent cyclization [146].

In 2009, Müller and co-workers introduced an alternative approach for synthesizing alkynones through a one-pot process [145]. Azulen-3-ylalkynones **126**, obtained by glyoxylation of azulene (**122**) with oxalyl chloride (**123**) and a subsequent decarbonylating Sonogashira coupling of glyoxylchloride **125**, were transformed in the same reaction vessel with methylhydrazine afforded pyrazoles **124** in moderate yields (Scheme 44) [146].

Analogous to Bishop's observations [73], a single regioisomer was isolated. However, attempts to carry out the cyclization with phenylhydrazine, Boc-hydrazine, or hydrazine hydrate failed.

**Scheme 45:**
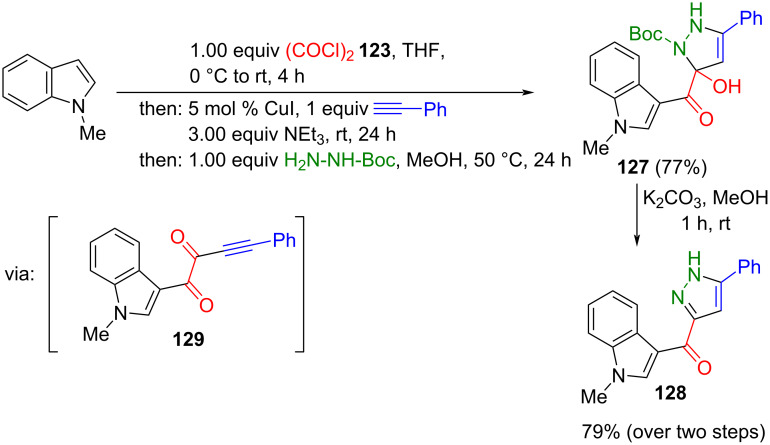
Four-component synthesis of a 3-indoloylpyrazole **128** [147].

Alternatively, alkynylation can be achieved through copper catalysis. Glyoxylation of *N*-methylindole with oxalyl chloride and subsequent Stephens–Castro coupling with phenylacetylene gives access to polyfunctional ynedione **129** [147]. In contrast to Sonogashira alkynylation, where glyoxyl substrates furnish ynones with concomitant decarbonylation [145], Stephens–Castro alkynylation preserves the dicarbonyl functionality in the product [148]. Embedded within a consecutive four-component reaction, with a concluding cyclocondensation employing Boc hydrazine, 1,5-diacyl-5-hydroxypyrazoline **127** is formed, as later confirmed by isolation [149]. Upon alkaline workup, this gives 3-acylpyrazole **128** in good yield (Scheme 45) [147].

**Scheme 46:**
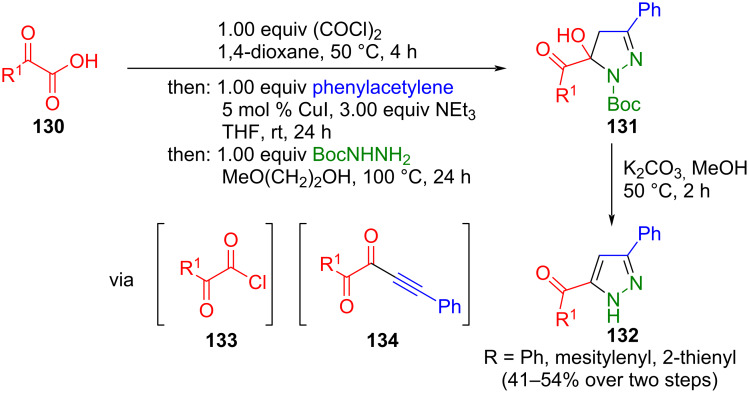
Two-step synthesis of 5-acylpyrazoles **132** via glyoxylation-Stephen–Castro sequence and subsequent cyclization with Boc hydrazine [148–149].

Ynediones are also accessible from α-ketocarboxylic acids **130** through chlorination and Stephens–Castro coupling [148]. With 1,4-dioxane as a solvent, chlorination yields could be increased by activation of oxalyl chloride to give glyoxyl chlorides **133**. The resulting ynediones **134** were cyclized in a consecutive three-component fashion with Boc-hydrazine to give 1,5-diacyl-5-hydroxypyrazolines **131**. Cleavage of the protecting group with potassium carbonate in methanol finally provides the corresponding 5-acyl NH-pyrazoles **132** (Scheme 46) [149].

**Scheme 47:**
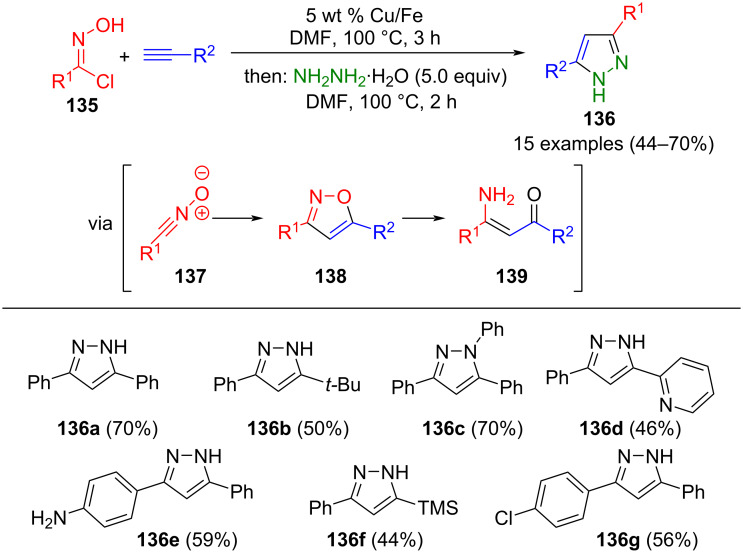
Copper on iron mediated consecutive three-component synthesis of 3,5-substituted pyrazoles **136** [150].

A novel approach to synthesizing pyrazoles via the initial formation of isoxazoles **138** through (3 + 2)-cycloaddition of nitrile oxides **137**, generated in situ from hydroxyiminoyl chloride **135** and terminal alkynes, was proposed by Kovacs and Novak. Copper supported on iron serves as a catalyst and as a reagent for the reductive ring opening and leads to β-aminoenones **139**, which react in the consecutive one-pot process with hydrazine hydrate to give 3,5-substituted pyrazoles **136** (Scheme 47) [150]. Notably, nitro groups are reduced to amines due to the reductive conditions. Furthermore, neither aliphatic hydroxyiminoyl chlorides nor internal alkynes are competent substrates in this transformation.

**Scheme 48:**
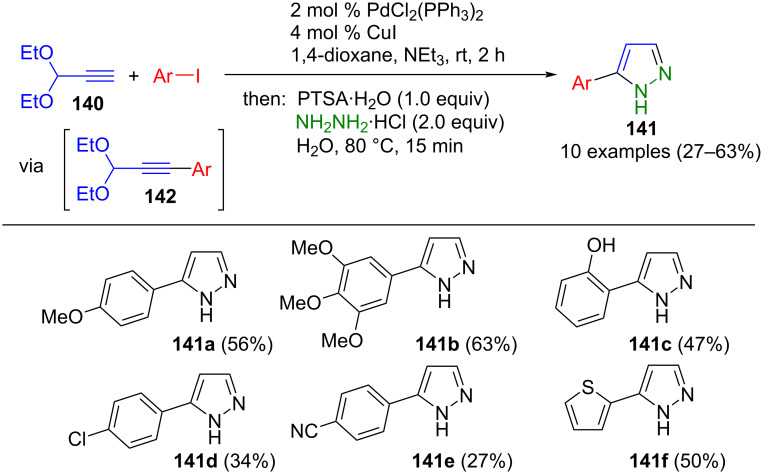
Consecutive three-component synthesis of 3-substituted pyrazoles **141** by Sonogashira coupling and subsequent cyclization with hydrazine hydrate [151].

Palladium catalysis not only enables the synthesis of alkynones, but also facilitates the introduction of protected alkynal moieties, which can be converted to pyrazoles in a one-pot fashion. Sonogashira coupling is particularly well-suited for this purpose. The alkynylation of propynal diethyl acetal (**140**) and (hetero)aryl iodides gives rise to 3-arylalkynyl acetals **142**. Since 3-arylpropynals are sensitive to oligo- and polymerization, it proved useful to perform acetal deprotection and cyclization with hydrazine hydrate in a one-pot procedure to give 3-substituted pyrazoles **141** (Scheme 48) [151]. Given that Sonogashira coupling is conducted under basic conditions, it is important to remove the protecting group with stoichiometric amounts of PTSA in this consecutive three-component synthesis. The process tolerates both electron-rich and electron-poor aryl substituents. However, it is not possible to introduce heteroaryls other than thiophene under standard conditions.

**Scheme 49:**
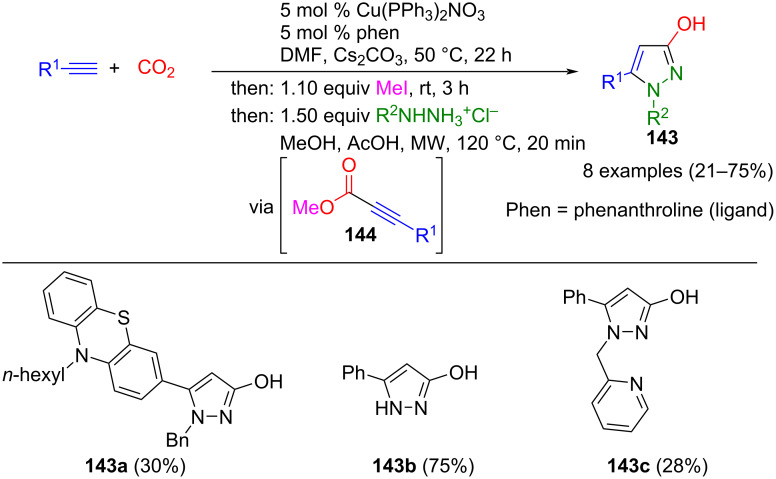
Consecutive three-component synthesis of pyrazoles **143** initiated by Cu(I)-catalyzed carboxylation of terminal alkynes [152].

Schreiner et al. established a further protocol for the one-pot synthesis of pyrazoles, where the alkynoyl moiety is generated by copper-catalyzed carboxylation of terminal alkynes followed by alkylation with methyl iodide, forming propiolic acid methyl esters **144**. Subsequently, these esters are converted to various 3-hydroxypyrazoles **143** with hydrazine salts by microwave-assisted cyclization (Scheme 49) [152].

The method tolerates a large number of functional groups. In addition, X-ray structural analysis proved that the products are aromatic 3-hydroxypyrazoles **143** and not tautomeric 3-pyrazolones.

**Scheme 50:**
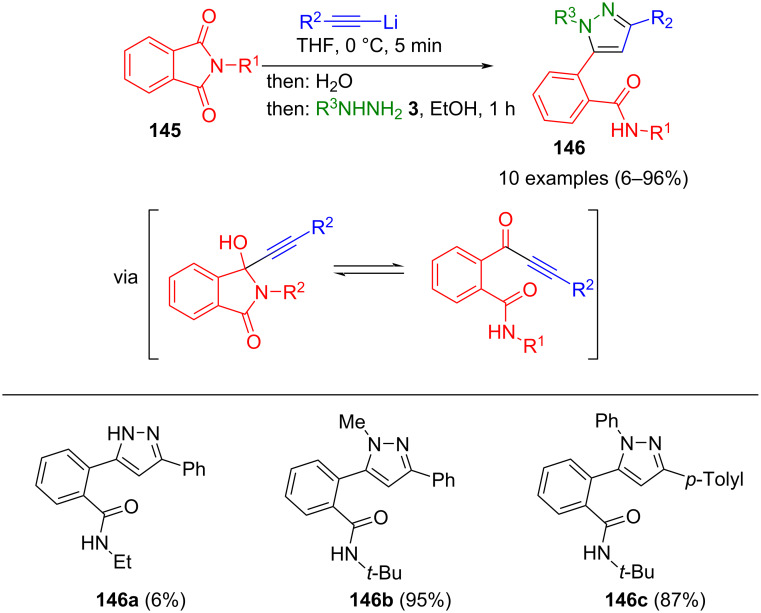
Consecutive three-component synthesis of benzamide-substituted pyrazoles **146** starting from *N*-phthalimides and lithiated alkynes [153–154].

Alternative access to alkynones can be achieved by nucleophilic addition of lithiated alkynes to *N*-substituted phthalimides **145**, followed by ring opening to give, upon the addition of water, the corresponding 3-hydroxyindolines. These intermediates are in equilibrium with the ring-opened alkynones. The latter reacts in a one-pot fashion with hydrazines to give pyrazoles **146** (Scheme 50) [153–154]. Notably, due to steric reasons, the cyclocondensation proceeds regioconvergently with methylhydrazine and phenylhydrazine.

Harigae et al. have presented a consecutive three-component synthesis of 3,5-disubstituted pyrazoles. This synthesis is initiated by adding terminal acetylides to aldehydes, followed by oxidation to the ynone utilizing molecular iodine, and concludes through cyclocondensation with hydrazines [155].

**Scheme 51:**
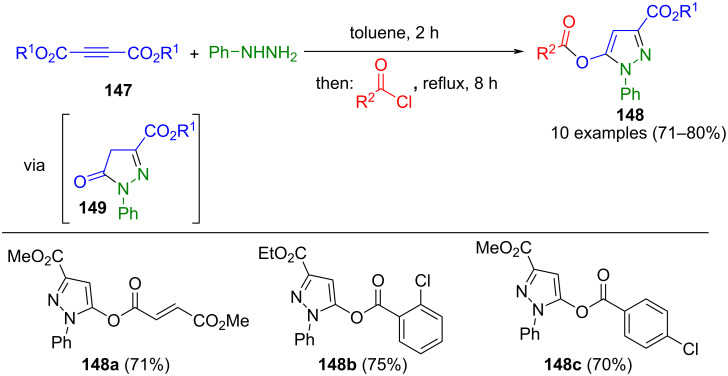
Consecutive three-component synthesis of 1,3,5-substituted pyrazoles **148** [156].

In line with the discussion on the formation of alkenone intermediates, dialkyl acetylenedicarboxylates **147**, as alkynoyl derivatives, are transformed to pyrazolones **149** by Michael addition–cyclocondensation with phenylhydrazine. In a consecutive one-pot reaction, the latter intermediates undergo *O*-acylation with aryl chlorides to give *N*-phenyl-3,5-substituted pyrazoles **148** (Scheme 51) [156]. Efforts to synthesize bridged pyrazoles using fumaryl chloride proved unsuccessful, with only 3,5-substituted pyrazoles isolated. Nonetheless, the crystal structure of such a pyrazole was investigated [157].

**Scheme 52:**
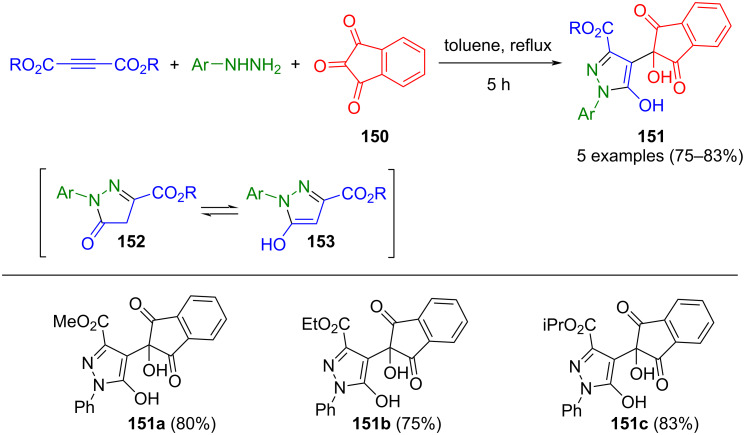
Three-component synthesis of 4-ninhydrin-substituted pyrazoles **151** [158].

In their study, Alizadeh et al. showed that ninhydrin (**150**), as an electrophile, reacts with the enol tautomeric form **153** of pyrazolone **152** to give ninhydrin-substituted pyrazoles **151** in a three-component reaction, as depicted in Scheme 52 [158].

**Scheme 53:**
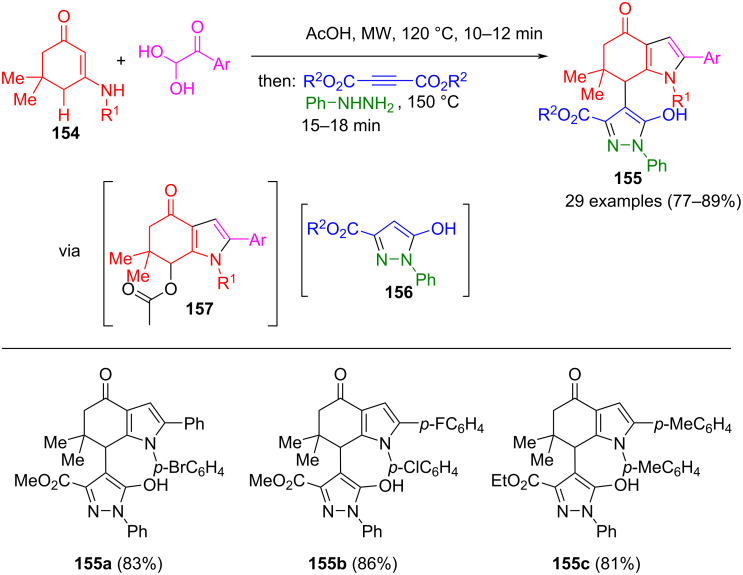
Consecutive four-component synthesis of 4-(oxoindol)-1-phenylpyrazole-3-carboxylates **155** [159].

The two-component formation of pyrazolone **156** from acetylenedicarboxylates and phenylhydrazine can be well embedded in en route formation of intermediate **157** from arylglyoxal and cyclic enaminone **154**. This transformation is catalyzed by acetic acid, ultimately yielding 4-(oxoindol)-1-phenylpyrazol-3-carboxylates **155** in the consecutive four-component process (Scheme 53) [159]. In this concept, the yields could be significantly increased by controlling the order of addition of reactants. It is noteworthy that both electron-rich and electron-poor arylglyoxals and enaminones lead to high yields in the reaction sequence.

**Scheme 54:**
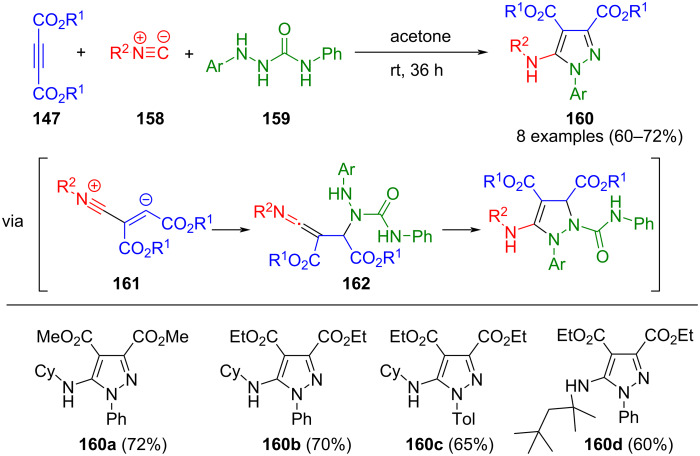
Three-component synthesis of pyrazoles **160** [160].

Abid et al. developed a concise three-component synthesis method for pyrazole starting materials from isonitriles **158**, dialkyl acetylenedicarboxylate **147**, and hydrazine carboxamide **159**. The addition of the isonitrile to the Michael system yields a nitrilium-vinyl anion zwitterion **161**, that is protonated by the hydrazine carboxamide, which undergoes addition to furnish the intermediary ketenimine **162**. The latter undergoes cyclization with elimination to form the corresponding pyrazoles **160** in a one-pot fashion (Scheme 54) [160]. The reaction can be extended by synthesizing hydrazone carboxamides in situ from hydrazine and isocyanates [161].

**Scheme 55:**
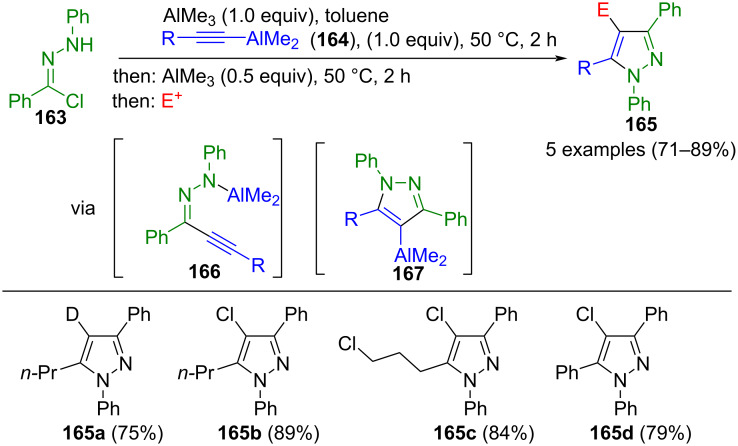
Consecutive three-component synthesis of pyrazoles **165** [162].

An unusual modification of alkynones is the use of aluminum alkynyl hydrazonides **166**, which are formed by nucleophilic displacement of hydrazonyl chlorides **163** with aluminum acetylides **164**. Subsequently, alkynyl hydrazonides **166** undergo metallacyclization to give aluminated pyrazoles **167**. These pyrazoles are then subjected to trapping with electrophiles, such as deuteration or electrophilic chlorination using *N*-chlorosuccinimide, in this consecutive three-component synthesis to give persubstituted pyrazoles **165** (Scheme 55) [162].

### (3 + 2)-Cycloaddition – C_2_ building blocks as substrates

1,3-Dipolar cycloadditions are important reactions in heterocycle synthesis [163–164] and certainly also for substituted pyrazoles. Four classes of 1,3-dipoles are considered as synthetic equivalents for the CN_2_ synthon: diazo compounds [165], nitrilimines [166], sydnones [167], and azomethinimines [168]. These intermediates undergo (3 + 2) cycloadditions with alkenes or alkynes to form pyrazoles, also in the sense of MCR. Furthermore, while not strictly classified as pericyclic reactions, hydrazones are also recognized as CN_2_ building blocks in pyrazole synthesis [169].

#### 1,3-Dipoles as key intermediates

Various methods have been developed for the in situ preparation of diazo compounds, as the substances are difficult to handle and toxic [170]. Approaches such as nitrogen transfer with tosylhydrazones, use of the Bestmann–Ohira reagent, or transformation of primary amines or azides, among others, represent viable and practical options.

**Scheme 56:**
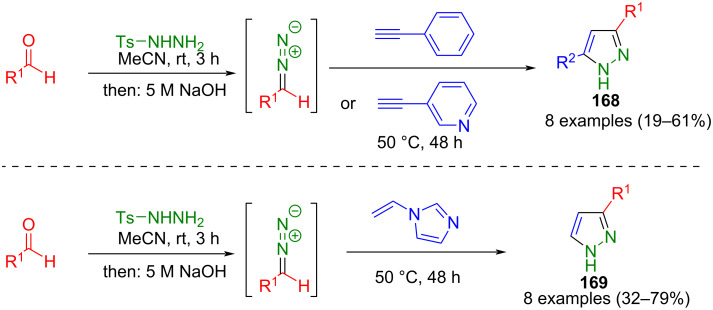
Consecutive three-component synthesis of 3,5-disubstituted and 3-substituted pyrazoles **168** and **169** from in situ formed diazo compounds [172].

Diazo compounds can be formed by basic treatment of intermediary tosylhydrazones, known from the Bamford–Stevens reaction [171]. Based on this, Aggarwal et al. developed a consecutive three-component synthesis of 3-substituted and 3,5-disubstituted pyrazoles **168** and **169** (Scheme 56) [172]. Tosylhydrazones are formed in situ from aromatic aldehydes and tosylhydrazine. After basic treatment of these tosylhydrazones followed by 1,3-dipolar cycloaddition with terminal alkynes, the corresponding pyrazoles **168** are obtained. The regioselectivity of this synthesis can be explained by steric causes and the favored HOMO (diazo compound)–LUMO (alkyne) interaction during the 1,3-dipolar cycloaddition [163], Wu et al. showed that electronic effects in this strategy do not influence the yield. In addition, sterically demanding reactants could be used in the method. Enhanced yields were achieved by using NaOEt and toluene [173]. *N*-Vinylimidazole, an alkene with a leaving group, was used to synthesize the 3-substituted pyrazoles **169** because, unlike acetylene, it is not gaseous and, therefore, easier to handle.

**Scheme 57:**
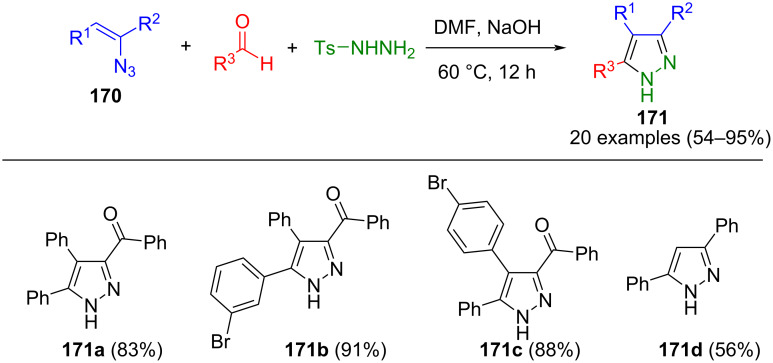
Three-component synthesis of 3,4,5-substituted pyrazoles **171** via 1,3-dipolar cycloaddition of vinylazides and in situ formed diazo compounds [174].

Instead of vinylimidazole, vinyl azides **170** can also be used as alkyne surrogates. After the 1,3-dipolar cycloaddition, the corresponding pyrazoles are formed by elimination of the azide group and subsequent tautomerization. Thus, the process enables access to 3,5- and 3,4,5-substituted pyrazoles **171** and also allows the synthesis of pyrazole 3-carboxylates (Scheme 57) [174]. An alternative method for the preparation of these pyrazoles involves the cycloaddition of diazo compounds generated in situ with (*Z*)-2-arylidene-2*H*-benzofuran-3-ones. This reaction yields intermediate spiropyrazolines, which react undergo 1,3-prototropic rearrangement to form the corresponding pyrazoles [175].

**Scheme 58:**
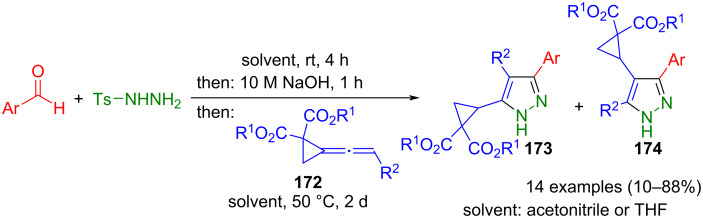
Three-component synthesis of pyrazoles **173** and **174** from aldehydes, tosylhydrazine, and vinylidene cyclopropane diesters **172** [176].

Aggarwal's method was also applied to synthesize 3,4,5-substituted pyrazoles **173** and **174** via 1,3-dipolar cycloaddition of in situ generated diazo compounds and vinylidenecyclopropane diesters **172**, which are synthetic equivalents of alkynes (Scheme 58) [176]. Notably, employing aromatic vinylidenecyclopropane diesters (R^2^ = aryl) in the method regioisomers **174** are selectively formed, while unsubstituted diesters (R^2^ = H) lead to pyrazoles **173** and **174** in a 1:2 ratio.

**Scheme 59:**
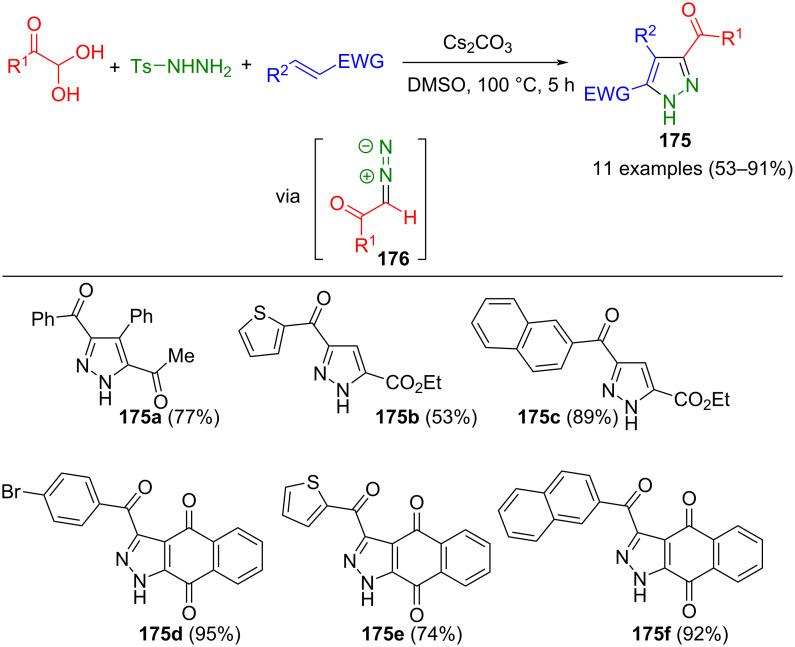
Three-component synthesis of pyrazoles **175** from glyoxyl hydrates, tosylhydrazine, and electron-deficient alkenes [177].

The aforementioned method can be varied by generating β-diazoketones **176** from arylglyoxals and tosylhydrazine, thus making pyrazole 5-carboxylates accessible. Shu et al. used this for the regioselective one-pot synthesis of pyrazoles **175** by 1,3-dipolar cycloaddition with electron-deficient alkenes (Scheme 59) [177]. The reaction is carried out in air to oxidize an intermediary pyrazoline. It tolerates a large number of substituents without affecting the yields. Notably, the use of vinylamides does not lead to the corresponding products. In addition to electron-poor alkenes, quinones and coumarins can also be used as reactants.

**Scheme 60:**
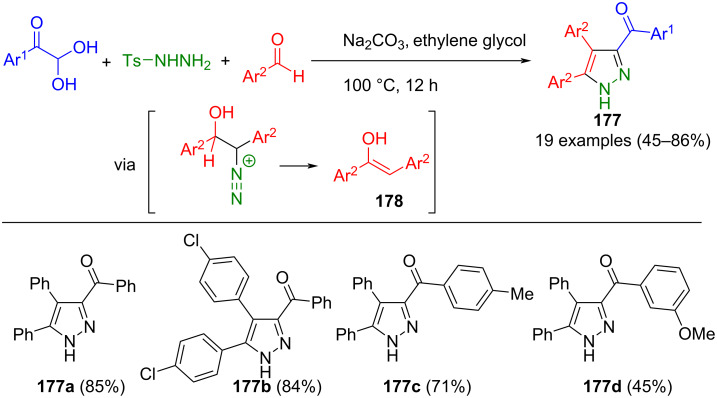
Pseudo-four-component synthesis of pyrazoles **177** from glyoxyl hydrates, tosylhydrazine, and aldehydes [178].

However, aldehydes can also be reacted in a domino reaction with in situ generated β-diazoketones. Initially, they condense with tosylhydrazine to form an enol **178** after basic treatment with a further equivalent of aldehyde. This reacts with β-diazoketones, formed from tosylhydrazine and arylglyoxals, in a 1,3-dipolar cycloaddition to give the corresponding pyrazole **177** (Scheme 60) [178]. In addition to aldehydes, ketones can also be used in the reaction. Moreover, it was observed that neutral and halogenated substituents on the arylglyoxal moiety lead to increased yields. However, a notable limitation of this method is its incompatibility with aliphatic aldehydes, likely due to the instability of the intermediary diazo compounds formed.

**Scheme 61:**
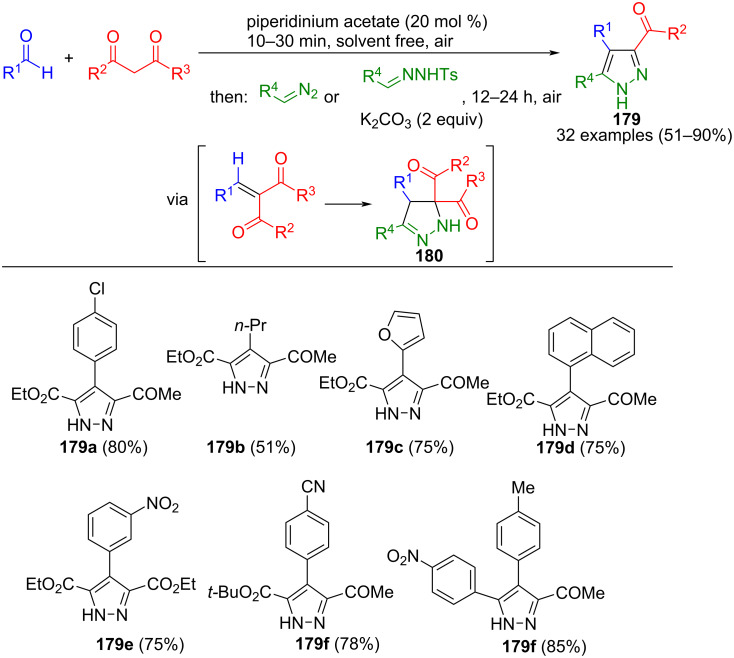
Consecutive three-component synthesis of pyrazoles **179** via Knoevenagel-cycloaddition sequence [179].

Kamal et al. also developed a consecutive three-component reaction for the synthesis of pyrazole 5-carboxylates **179** (Scheme 61) [179]. In contrast to the previously mentioned methods, however, tosylhydrazones were directly employed. In the concept, unsaturated carbonyl compounds are initially formed via Koevenagel condensation between aldehydes and 1,3-carbonyl compounds. These intermediates then undergo reaction under basic conditions with tosylhydrazones to yield 3-diacylpyrazolines **180**. Subsequent elimination of the acyl group facilitated by water and potassium carbonate furnishes the corresponding pyrazoles after aerobic aromatization. In the method, aromatic aldehydes with electron-withdrawing substituents demonstrate enhanced yields. In addition, both ketones and β-ketoesters are tolerated in the sequence. Aromatic hydrazones and β-carboxyhydrazones can be used as CN_2_ building blocks. It was also shown that the process can be carried out on a gram scale and that diazoalkanes can also be used as starting materials in addition to tosylhydrazones.

**Scheme 62:**
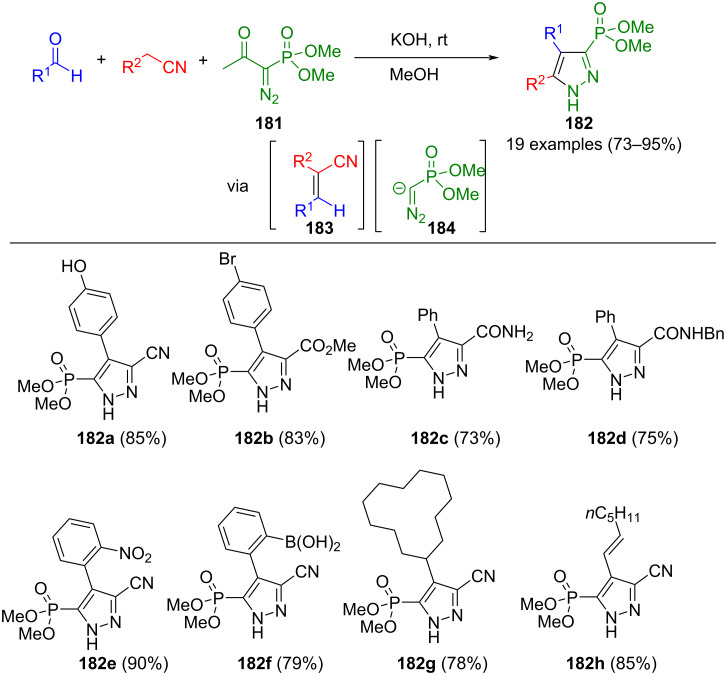
Three-component synthesis of 5-dimethylphosphonate substituted pyrazoles **182** from aldehydes, the Bestmann–Ohira reagent (**181**), and nitriles [180].

Another potent source of diazoalkane is the Bestmann–Ohira reagent (**181**). Upon basic treatment, the Seyferth–Gilbert reagent is generated through acyl cleavage, which has been converted to pyrazoles via various reactions. In a domino three-component reaction, unsaturated nitriles **183** are initially produced from aldehydes and nitriles in a Knoevenagel reaction. These undergo a formal 1,3-dipolar cycloaddition with the intermediary Seyferth–Gilbert reagent (**184**), followed by the elimination of hydrogen cyanide to give 5-phosphonylpyrazoles **182** (Scheme 62) [180]. The reaction sequence tolerates a diverse range of substituents, and electronic effects have no influence on the yields. Only very weakly activated nitriles do not lead to the desired product. Remarkably, no homologation of the aldehyde to the alkyne takes place during the reaction due to the faster formation of the unsaturated nitriles, which supersedes possible side reaction. The present method can be extended to include a CuAAC reaction using alkyne-functionalized aldehydes, thus enabling the synthesis of interesting molecules pertinent to pharmaceutical and agrochemical applications.

**Scheme 63:**
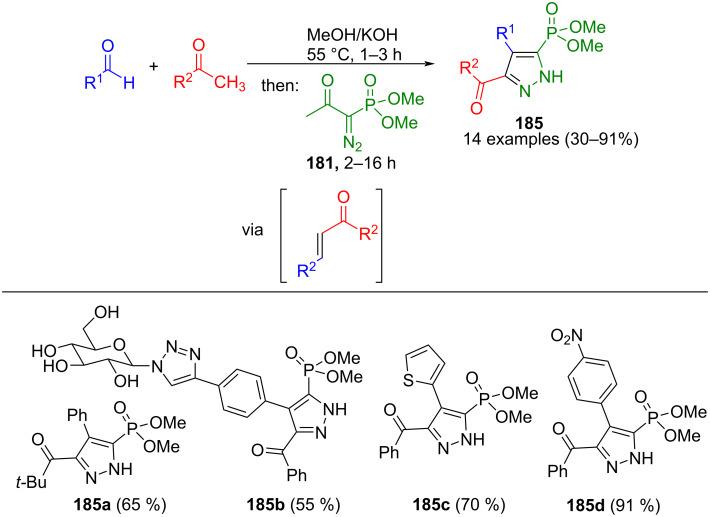
Consecutive three-component synthesis of 5-(dimethyl phosphonate)-substituted pyrazoles **185** from aldehydes, methyl ketones, and the Bestmann–Ohira reagent (**181**) via Claisen–Schmidt/1,3-dipolar cycloaddition/oxidation sequence [181].

The same research group also showed another way to prepare 5-phosphonylpyrazoles **185** (Scheme 63) [181]. Through a Claisen–Schmidt/1,3-dipolar cycloaddition/oxidation sequence, the aforementioned pyrazoles were obtained from methyl ketones, aldehydes, and the Bestmann–Ohira reagent (**181**). Both the use of aromatic aldehydes with electron-withdrawing substituents and electron-poor methyl ketones increase the yields. Steric effects play a crucial role in this method, as *ortho*-substituents lead to steric hindrance of the α,β-unsaturated ketone, thus impeding pyrazole formation. Nonetheless, a notable advantage of this process is that it can be combined with the CuAAC reaction.

**Scheme 64:**
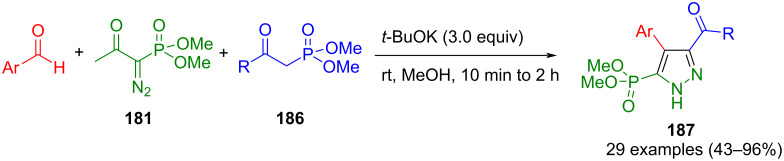
Three-component synthesis of 5-(dimethyl phosphonate)-substituted pyrazoles **187** from aldehydes, the Bestmann–Ohira reagent (**181**), and β-keto phosphonates **186** [182].

Another pyrazole synthesis using the Bestmann–Ohira reagent (**181**) was reported by Ahamad and co-workers. Enones can be synthesized in situ from β-ketophosphonates **186** and aldehydes by Horner–Emmons–Wadsworth olefination. These enones undergo cyclization with the Seyferth–Gilbert reagent, forming the corresponding pyrazole **187** upon oxidation. Notably, if the method is carried out as a domino reaction, the yields are higher than with a stepwise addition of the reactants (Scheme 64) [182]. In addition to the Bestmann–Ohira reagent, diazosulfones can also be used in the process. Here, 3,4-disubstituted pyrazoles are formed in a Horner–Emmons–Wadsworth/1,3 dipolar cycloaddition/desulfonation process [183].

**Scheme 65:**
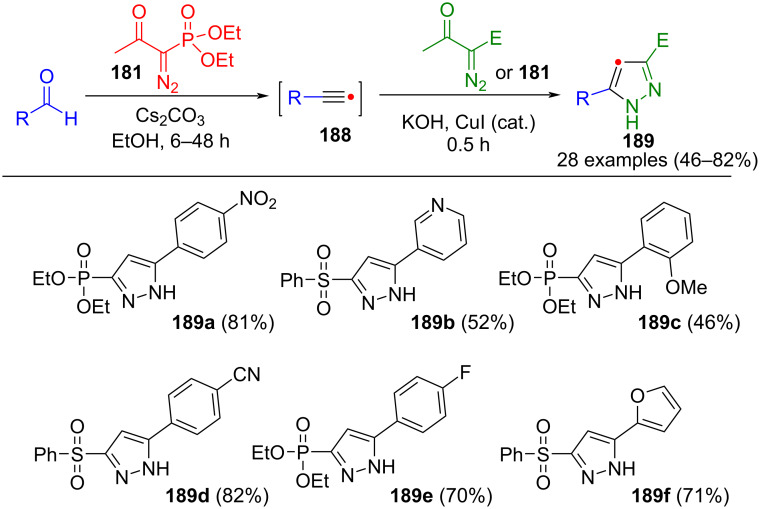
Three-component synthesis of 5-diethylphosphonate/5-phenylsulfonyl substituted pyrazoles **189** from aldehydes, Bestmann–Ohira reagent (**181**), and α-diazo-β-keto esters [184].

The Bestmann–Ohira reagent (**181**) itself can also be used as a Horner–Emmons–Wadsworth reagent for the homologation of aldehydes to generate alkynes **188** in situ. Kumar et al. took advantage of this and also used the Bestmann–Ohira reagent as a cycloaddition partner. Pyrazoles **189** were obtained under basic conditions in a consecutive three-component reaction (Scheme 65) [184]. In the method, copper iodide must be added to activate the alkynes and the Seyferth–Gilbert reagent, as the intermediates are not sufficiently reactive. Moreover, by varying the starting materials, additionally, 5-sulfonylpyrazoles can be produced using this method.

**Scheme 66:**
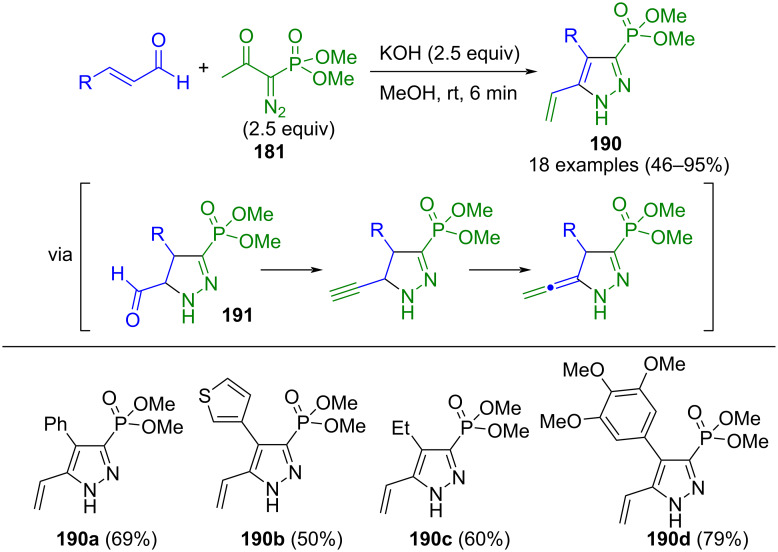
Pseudo-three-component synthesis of 3-(dimethyl phosphonate)-substituted pyrazoles **190** [185].

In reactions involving α,β-unsaturated aldehydes, the Bestmann–Ohira reagent (**181**) can also take on a dual role. The Seyferth–Gilbert reagent generated from this engages in a cycloaddition with the vinyl unit, yielding a pyrazolinecarboxaldehyde **191**. Subsequent treatment with another equivalent of **181** homologates the intermediate’s aldehyde moiety, giving the corresponding pyrazole **190** after 1,3-hydrogen shift and subsequent aromatization (Scheme 66) [185]. The reaction proceeds independently of the substituents of the aldehyde and tolerates electron-withdrawing and electron-donating substituents.

**Scheme 67:**
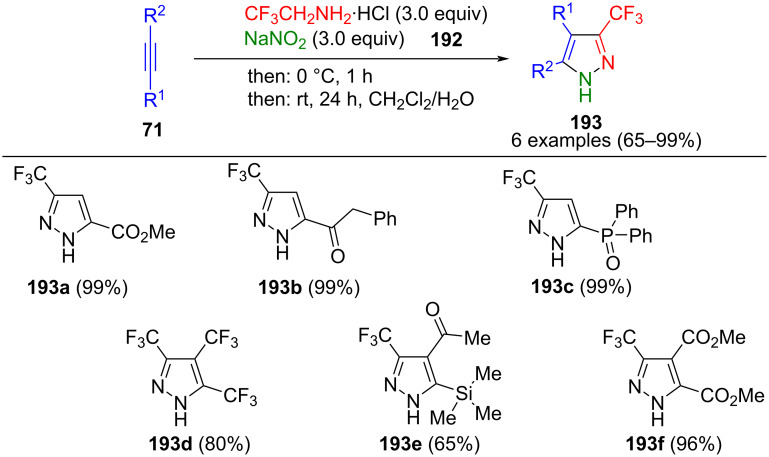
Three-component synthesis of 3-trifluoromethylpyrazoles **193** [186].

Slobodyanyuk et al. developed a one-pot method for the synthesis of 3-trifluoromethylpyrazoles **193**, in which a diazo compound was synthesized in situ from sodium nitrite and CF_3_CH_2_NH_2_·HCl **192** (Scheme 67) [186]. The reaction with alkynes with at least one electron-withdrawing substituent leads to the corresponding pyrazoles. Notably, the reaction is regioselective, and only 3,5-substituted pyrazoles are formed. However, internal alkynes can also partake in the reaction, provided that at least one electron-withdrawing group is present. If the second substituent is also electron-withdrawing, the reaction proceeds in excellent yields. The reaction time has to be extended for electron-rich substituents, whereas strongly electron-releasing substituents fail to yield the desired product. Surprisingly, an inverse regioselectivity was observed for reaction of the diazoalkane with a TMS-substituted internal alkyne, presumably due to steric hindrance. Since the reactants only react in a specific order, they can all be present initially. In addition, the reaction can be scaled up to 100 g. The reaction of terminal alkynes can be catalyzed with Ag_2_O [187]. In addition to alkynes, alkynones are also tolerated in this cycloaddition [188]. The process can also be carried out in a continuous flow reactor and can be extended by *N*-methylation, Chan–Lam–Evans coupling, or amidation of the corresponding substituents [189–190].

**Scheme 68:**
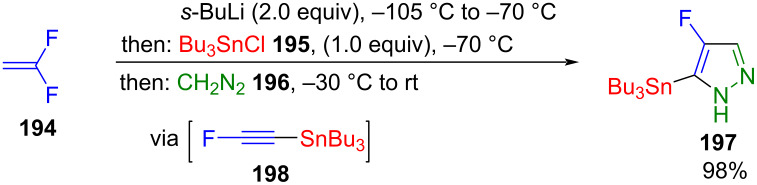
Consecutive three-component synthesis of 5-stannyl-substituted 4-fluoropyrazole **197** [191–192].

Hanamoto et al. succeeded in synthesizing 5-tributylstannyl-4-fluoropyrazole (**197**) using one-pot methodology. This compound was formed from diazomethane (**196**) and an in situ generated alkyne **198**. After treatment with butyllithium and reaction with chlorotributylstannane (**195**), 1,1-difluoroethene (**194**) was used as an intermediate to generate **198**. This alkyne immediately formed the corresponding pyrazole **197** in a 1,3-dipolar cycloaddition with diazomethane (Scheme 68) [191–192]. Notably, the synthesis of pyrazoles **197** is regioselective due to the HOMO–LUMO interaction of the 1,3-dipolar cycloaddition [163]. Additionally, the reaction can be alternatively conducted using chlorotrimethylsilane instead of chlorotributylstannane [193]. A variation with bis(tributyltin) oxide and 3,3,3-trifluoro-2-bromopropene is also possible [194].

**Scheme 69:**
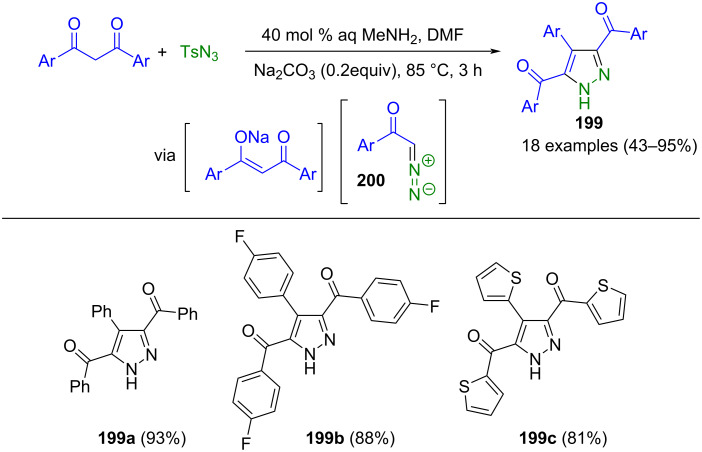
Pseudo-three-component synthesis of 3,5-diacyl-4-arylpyrazoles **199** [195].

3,5-Diacyl-4-arylpyrazoles **199** can be prepared from aromatic 1,3-dicarbonyl compounds in a pseudo-multicomponent reaction (Scheme 69) [195]. From 1,3-dicarbonyl compounds, α-aryldiazomethanes **200** were generated in situ by Regitz diazo transfer and concomitant acyl cleavage with methylamine. Sodium carbonate facilitates the enolate formation of the 1,3-dicarbonyl compound. One limitation of the method is that *ortho*-substituents lead to reduced yields and that strongly electron-withdrawing substituents are not tolerated.

**Scheme 70:**
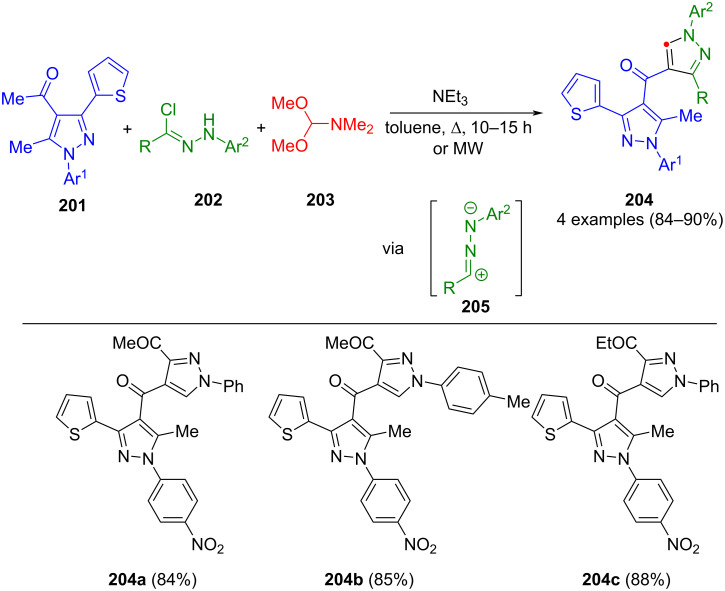
Three-component synthesis of pyrazoles **204** via nitrilimines [196].

Other important dipoles are nitrilimines **205**, which can be synthesized in situ by the basic treatment of hydrazonyl chlorides **202**. This concept was used by Gomha et al. in a domino reaction, wherein 4-acylpyrazoles **201** and DMF-dimethylacetal (**203**) initially form the corresponding enaminones, which subsequently engage in a cycloaddition with nitrilimines, giving bispyrazoles **204** (Scheme 70) [196]. The yields were increased by dielectric heating. Interestingly, one of the products presented (**204a**) shows inhibitory activity against hepatocellular carcinoma (HepG-2).

**Scheme 71:**
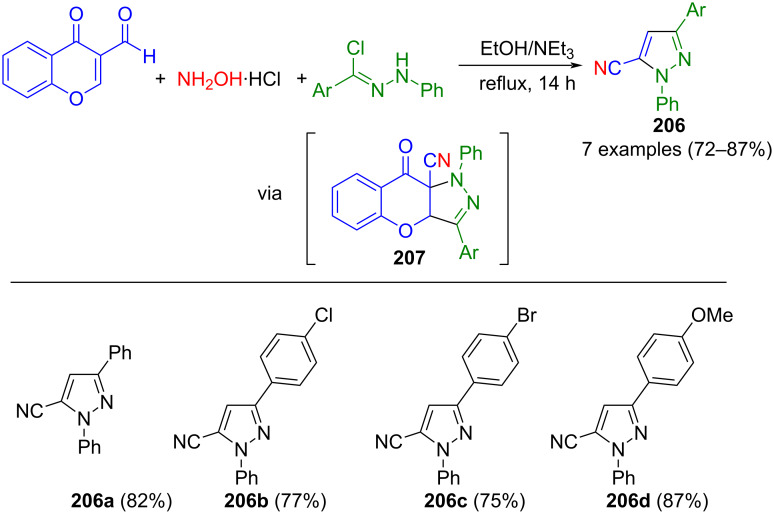
Three-component synthesis of 1,3,5-substituted pyrazoles **206** via formation of nitrilimines and salicylate elimination [197].

Alizadeh et al. also used nitrilimines in a three-component reaction to synthesize 1,3-substituted 5-cyanopyrazoles **206** (Scheme 71) [197]. First, the reaction between 4-oxo-4*H*-chromene-3-carbaldehyde and hydroxylamine yielded 4-oxo-4*H*-chromene-3-cyanide. Subsequently, in a 1,3-dipolar cycloaddition with in situ-generated nitrilimines, intermediate **207** was formed. A nucleophilic attack by ethanol and concomitant elimination of ethyl salicylicate lead to pyrazoles **206**. In the method, both electron-rich and electron-poor arylhydrazonyl chlorides lead to excellent yields.

**Scheme 72:**
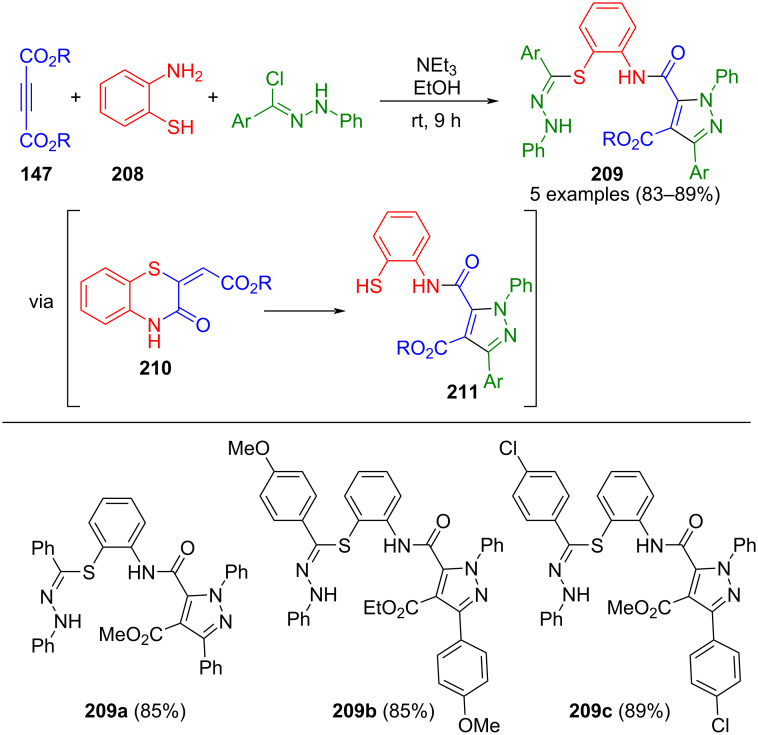
Pseudo four-component synthesis of pyrazoles **209** from acetylene dicarboxylates **147**, hydrazonyl chloride, and 2-aminothiophenol (**208**) [198].

In an alternative approach, the same research team used acetylenedicarboxylates **147** as two-carbon building blocks for pyrazole synthesis. Initially, a domino reaction was employed to form 1,4-benzothiazine-3(4*H*)-one **210** through the cyclocondensation of 2-aminothiophenol (**208**). Subsequently, in situ-generated nitrilimine from the hydrazonyl chloride undergoes a 1,3-dipolar cycloaddition, resulting in the formation of thiophenol **211** after ring opening. This thiophenol **211** then reacts with another equivalent of hydrazonyl chloride to furnish pyrazole **209** (Scheme 72) [198]. The products could be additionally corroborated by X-ray structure analysis.

**Scheme 73:**
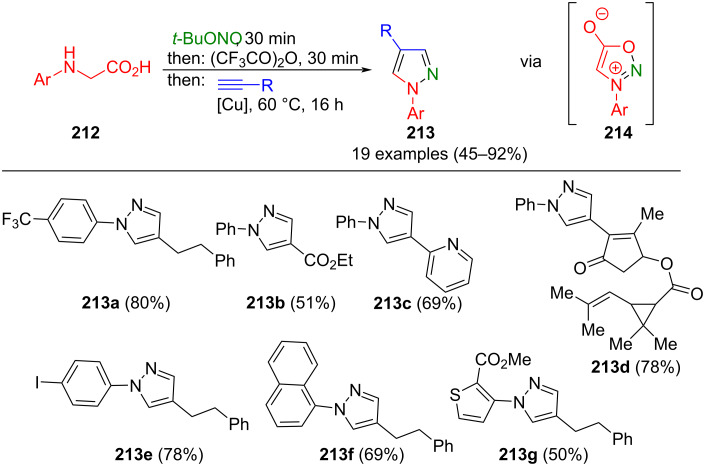
Consecutive three-component synthesis of pyrazoles **213** via syndnones **214** [200].

Sydnones **214** are mesomerically stabilized compounds that represent synthetic equivalents to diazomethinimes. In a copper-catalyzed sydnone-alkyne cycloaddition (CuSAC), they can be converted to pyrazoles with high efficiency and chemoselectivity [199]. Specklin et al. used this strategy for the consecutive three-component synthesis of 1,4-substituted pyrazoles **213** (Scheme 73) [200]. Sydnones are generated in situ by nitrosylation of arylglycines **212** with *t*-BuONO, followed by cyclization with trifluoroacetic anhydride. After adding terminal alkynes, the corresponding pyrazoles are formed via CuSAC reaction with subsequent decarboxylation. Notably, the process tolerates both electron-poor and electron-rich aryls, as well as numerous functional groups. It is noteworthy that the solvent THF leads to reduced yields, while *t*-BuOH/water proves to work well.

**Scheme 74:**
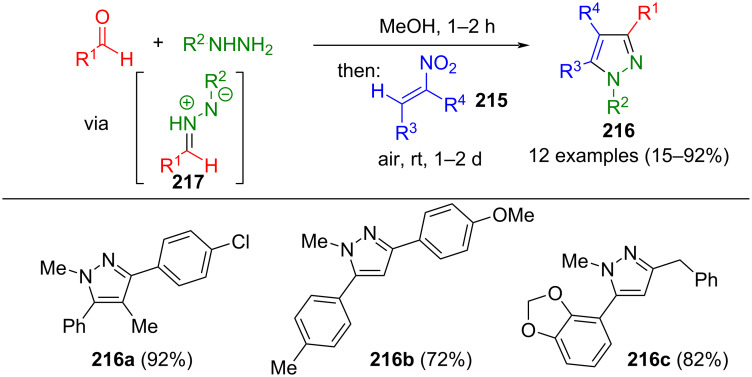
Consecutive three-component synthesis of pyrazoles **216** via in situ-formed diazomethinimines **217** [201].

Other useful 1,3-dipoles utilized in cycloaddition reactions are diazomethinimines **217**. In some reactions, they are formed from hydrazones, which are generated from the condensation of aldehydes and hydrazines. In a sequential three-component reaction, diazomethinimines undergo 1,3-dipolar cycloaddition with nitroolefins **215**, forming pyrazolines. Under aerobic conditions, these pyrazolines undergo gradual oxidation and are subsequently transformed into the corresponding pyrazoles **216** through rapid nitrite elimination (Scheme 74) [201].

During the reaction, the pyrazoline intermediate exists in equilibrium with its Michael adduct, which is incapable of cyclization. Using polar solvents, the equilibrium can be shifted towards the formation of pyrazoline due to the stabilization of the dipolar diazomethinimines. However, a limitation of this method arises with strongly electron-withdrawing aldehydes, as they lead only to Michael adducts. The present concept can also be used to synthesize 3-(pyrazol-5-yl)chromones if 3-nitrovinylchromones are chosen as a starting material [202].

**Scheme 75:**
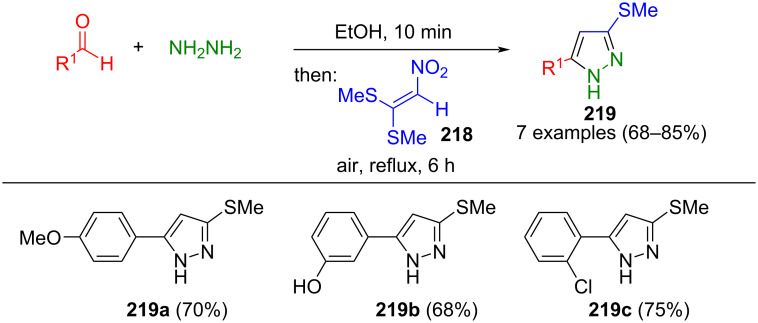
Consecutive three-component synthesis of 3-methylthiopyrazoles **219** from aldehydes, hydrazine, and 1,1-bis(methylsulfanyl)-2-nitroethene (**218**) [203].

Furthermore, this method was also applied to 1,1-bis(methylsulfanyl)-2-nitroethene (**218**) for the consecutive three-component synthesis of 3-methylthiopyrazoles **219** in boiling ethanol (Scheme 75) [203]. In a similar method, nitrilimines can also be synthesized from aldehydes and hydrazines after treatment with Hg(OAc)_2_ to react in a 1,3-dipolar cycloaddition with alkynes, affording the corresponding pyrazoles [204].

#### Hydrazones as key intermediates

The cyclization of hydrazones is similar to the 1,3-dipolar cycloaddition. Here, too, CN_2_ building blocks are formed first, which are then converted to pyrazoles. However, hydrazones preferably react with β-ketoesters or ketones. Alternatively, they can undergo cyclization with alkynes. However, no dipolar intermediates are involved in these reactions.

**Scheme 76:**
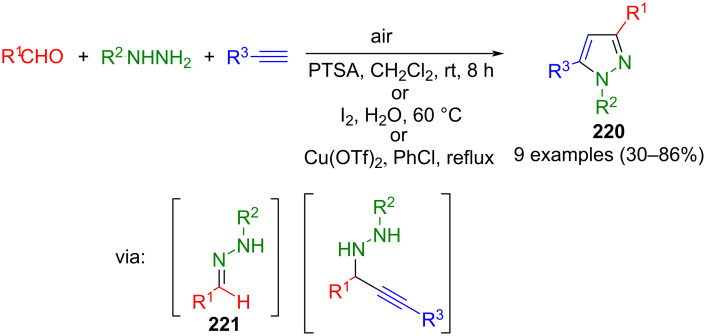
Three-component synthesis of 1,3,5-substituted pyrazoles **220** from aldehydes, hydrazines, and terminal alkynes [205–207].

For example, 1,3,5-substituted pyrazoles **220** can be synthesized via a domino Mannich/cyclization/oxidation reaction of terminal alkynes and hydrazones **221**, which are in situ generated from aromatic hydrazines and aldehydes (Scheme 76) [205]. This method tolerates a broad spectrum of functional groups, and using of electron-rich alkynes and aldehydes increases the yields. Multifunctional catalysts such as PTSA [205] and molecular iodine are viable options for catalyzing this sequence [206]. In addition, the process can be carried out with tosylhydrazine under copper catalysis [207].

**Scheme 77:**
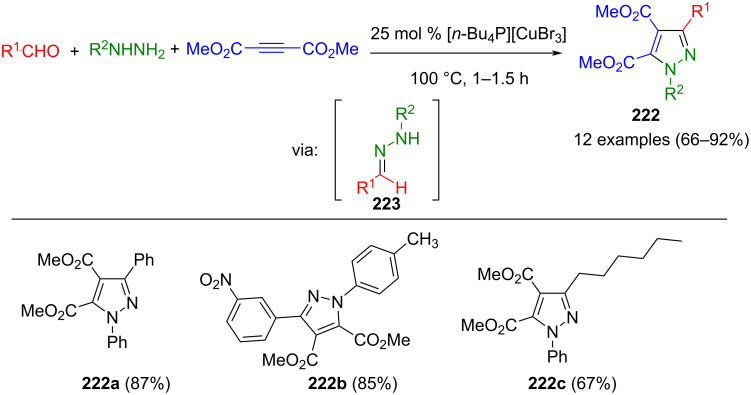
Three-component synthesis of 1,3,4,5-substituted pyrazoles **222** from aldehydes, hydrazines, and DMAD [208].

Internal alkynes must be used to access fully substituted pyrazoles from hydrazones. Dimethyl acetylenedicarboxylate (DMAD) is a useful building block for this purpose. In the ionic liquid [*n*-Bu_4_P][CuBr_3_], DMAD reacts strictly chemoselectively with hydrazones **223** formed in situ to give intermediary pyrazolines via radical cyclization. These are subsequently oxidized under an aerobic atmosphere to furnish 1,3,4,5-substituted pyrazoles **222** (Scheme 77) [208]. Since oxidation does not occur when very electron-rich aldehydes are used, these cannot be employed in this sequence. Besides simple aromatic aldehydes, 3-formylchromones are also tolerated in the reaction. The latter react with hydrazine in a one-pot process and form the corresponding pyrazoles with DMAD under Yb(OTf)_3_ catalysis [209].

**Scheme 78:**
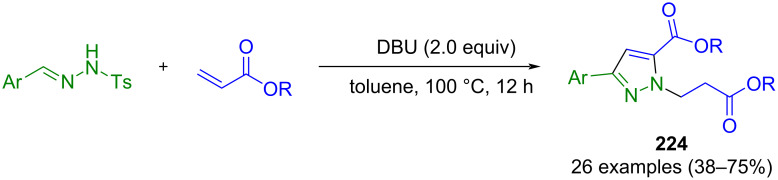
Pseudo three-component synthesis of pyrazoles **224** from sulfonyl hydrazone and benzyl acrylate under transition-metal-free conditions [210].

The research team led by Hao introduced a straightforward and novel approach for synthesizing pyrazoles **224** utilizing benzene sulfonylhydrazone and benzyl acrylate. This (3 + 2)-cycloaddition reaction simultaneously constructed two new C–N bonds and one C–C bond under transition-metal-free conditions (Scheme 78) [210]. The introduction of diverse electron-donating or -withdrawing groups at the *ortho*-, *para*- and *meta*-positions of benzene sulfonyl hydrazone demonstrates excellent compatibility within this transformation.

#### Miscellaneous processes

**Scheme 79:**
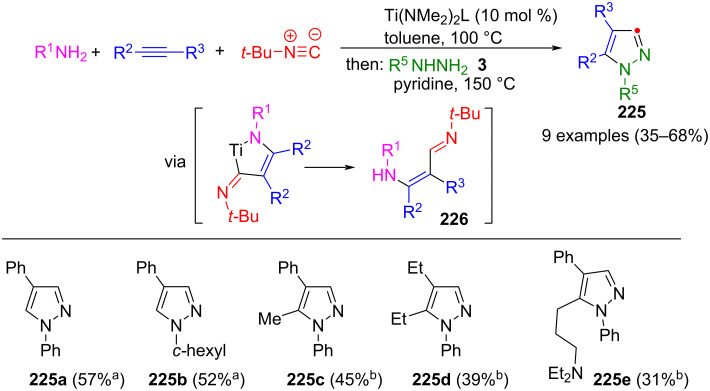
Titanium-catalyzed consecutive four-component synthesis of pyrazoles **225** via enamino imines **226** [211]. ^a^10 mol% Ti(NMe_2_)_2_(dpma); ^b^20 mol % Ti(dpm)(NMe_2_)_2_.

In addition to the methods discussed above, there have also been proposed several miscellaneous methods that do not fit into the framework of (3 + 2)-cyclocondensations or cycloadditions. In pyrazole synthesis, C–N bonds can also be formed by titanium catalysis. Majumder et al. developed a protocol for the consecutive four-component synthesis of 4,5-substituted pyrazoles, as only a few methods are known for this. Initially, 3-aminoimines **226** are synthesized from alkynes, primary amines, and *tert*-butyl isocyanide using titanium catalysis. Subsequently, these intermediates are cyclized in a consecutive multicomponent reaction with hydrazines to give 4,5-substituted pyrazoles **225** (Scheme 79) [211]. Notably, both terminal and internal alkynes are tolerated in the reaction, with the more active catalyst Ti(dpm)(NMe_2_)_2_ typically used for the latter. One limitation, however, is that a mixture of regioisomers is obtained when utilizing these alkynes, even if one is preferentially formed.

**Scheme 80:**
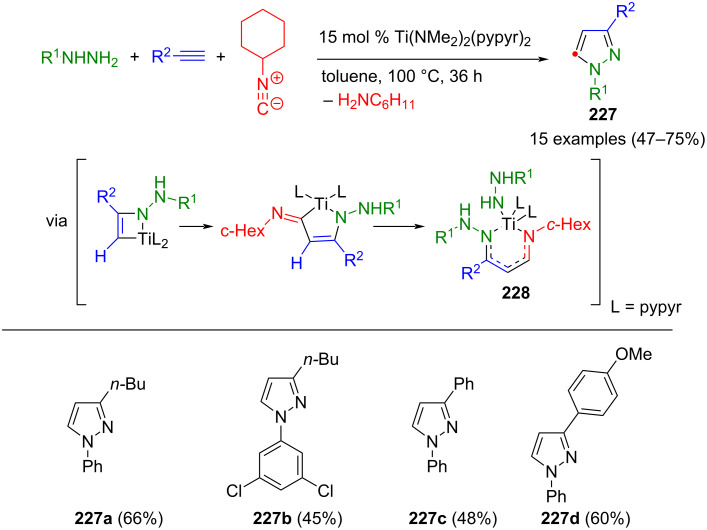
Titanium-catalyzed three-component synthesis of pyrazoles **227** via enhydrazino imine complex intermediates [212].

Using hydrazines instead of amines in the above reaction enables the synthesis of pyrazoles via a domino reaction. This involves a (2 + 2)-cycloaddition with terminal alkynes, followed by the α-insertion of isonitriles, and protonolysis of another equivalent of hydrazine with the metal, leading to six-membered intermediates **228**. Subsequent intramolecular cyclization leads to the formation of a pyrazoline, which, upon elimination of cyclohexylamine, forms the corresponding pyrazole **227** (Scheme 80) [212]. The (2 + 2)-cycloaddition exhibits regioselectivity due to steric effects. However, unlike the previously mentioned method, this approach yields 3-substituted pyrazoles.

**Scheme 81:**
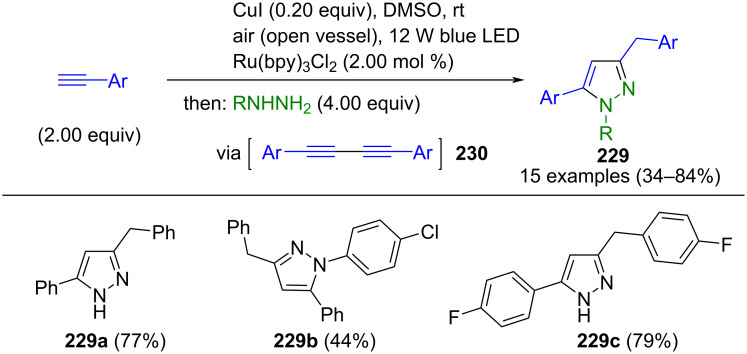
Pseudo-three-component synthesis of pyrazoles **229** via Glaser coupling of terminal alkynes and photocatalytic cyclization with hydrazines [214].

The copper-catalyzed Glaser coupling of alkynes enables concise access to symmetrical diynes **230** [213]. Concatenation via cyclization with hydrazines enables the pseudo-three-component synthesis of pyrazoles **229**, employing photoredox catalysis with blue LED lamps and ruthenium complexes as a catalyst system (Scheme 81) [214].

Interestingly, UV light is indispensable for both reaction steps of the sequence, while the ruthenium catalyst additionally accelerates the Glaser coupling. The reaction tolerates a broad spectrum of functional groups, and electron-withdrawing aryl substituents lead to increased yields. In addition, both methylhydrazine and phenylhydrazine can be used, while sterically demanding aliphatic hydrazines fail to yield the desired product.

**Scheme 82:**
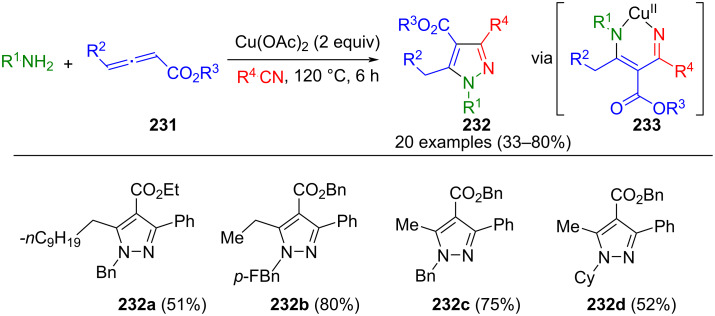
Copper(II)acetate-mediated three-component synthesis of pyrazoles **232** [216].

In addition to C–C and C–N bond formation, transition metal catalysis can also form N–N bonds. In their investigations into the intramolecular cyclization of enaminoates, Neumann et al. uncovered an interesting phenomenon. When the reaction was carried out in acetonitrile, enaminoates coupled with the nitrile solvent in an intramolecular oxidation, leading to pyrazoles [215]. Remarkably, this N–N bond formation is copper-mediated, where copper acts both as a Lewis acid activator of the nitrile and an oxidizing agent. Under similar conditions, primary amines, nitriles, and 2,3-allenoates **231** engage in a three-component reaction via enamino-imine complexes **233** to afford pyrazoles **232** (Scheme 82) [216]. The nitrile is present in excess as a solvent in this domino reaction. Furthermore, in addition to allenoates, 2-alkynoates can also be used as starting substrates.

**Scheme 83:**
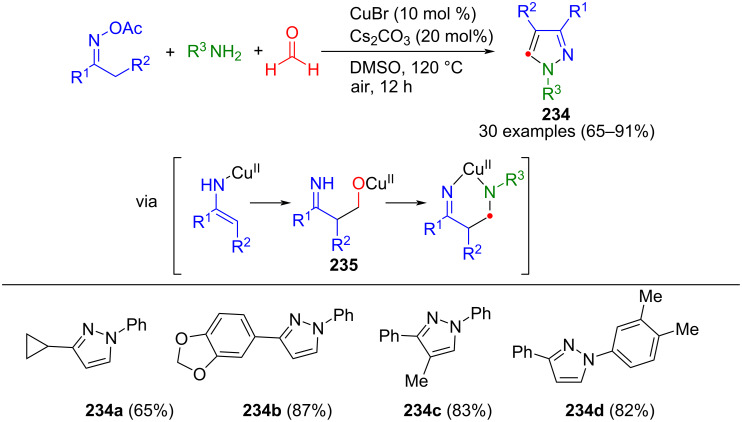
Copper-catalyzed three-component synthesis of 1,3,4-substituted pyrazole **234** from oxime acetates, anilines, and paraformaldehyde [217].

Tang et al. developed a catalytic variant for forming N–N bonds, as stoichiometric quantities of copper were used in the previously mentioned method. Imines **235** are generated from oxime acetates through copper(I)-mediated N–O bond scission, followed by nucleophilic addition of paraformaldehyde. Subsequent coupling with aniline and reductive elimination produces pyrazolines, which aromatize under aerobic conditions, to give pyrazoles **234** in a three-component reaction (Scheme 83) [217]. The introduction of caesium carbonate serves to enhance the reaction's conversion, although elevated base concentration leads to the deactivation of the oxime acetates. The method demonstrates tolerance towards both aliphatic and aromatic oxime acetates, with electron-rich substituents giving higher yields. A complementary observation is that anilines bearing electron-withdrawing substituents increase overall yields, whereas aliphatic amines are not tolerated in this method. A further limitation of the method is that the aldehyde reaction partner is restricted to paraformaldehyde.

**Scheme 84:**
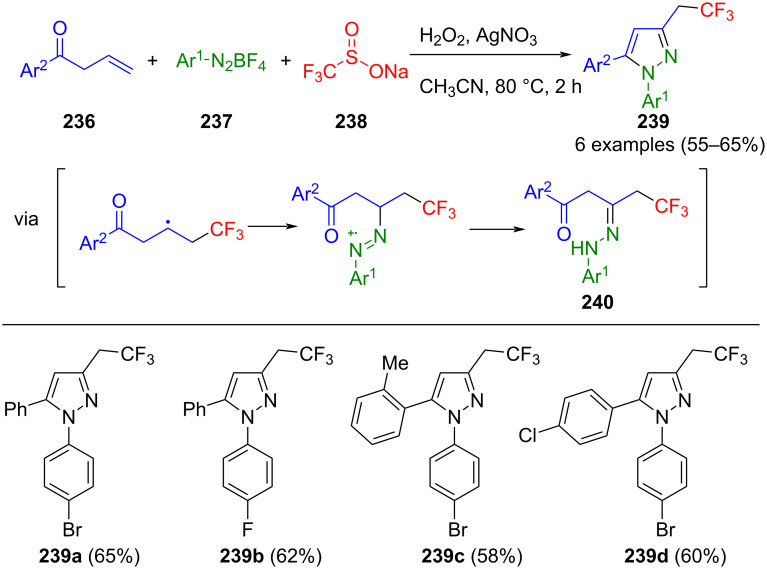
Three-component synthesis of 3-trifluoroethylpyrazoles **239** [218].

Pyrazoles can be prepared via a radical-mediated one-pot process. For example, 2,2,2-trifluoroethyl groups can be introduced to the pyrazole core. In a notable three-component synthesis (Scheme 84), enones **236** undergo a reaction with hydrogen peroxide, silver nitrate, CF_3_SO_2_Na (**237**), and aryldiazonium salts **238**, yielding pyrazole **239** [218]. After the addition of the trifluoromethyl radical and the diazonium salt, hydrazones **240** are formed, which subsequently undergo intramolecular cyclocondensation, giving the corresponding pyrazole **239**. Remarkably, the process tolerates various substituents on both the aryl and the arylallyl ketone. Furthermore, its regioselectivity represents an additional advantage. Notably, indoles and pyridazinones can be produced in a one-pot process using this concept.

**Scheme 85:**
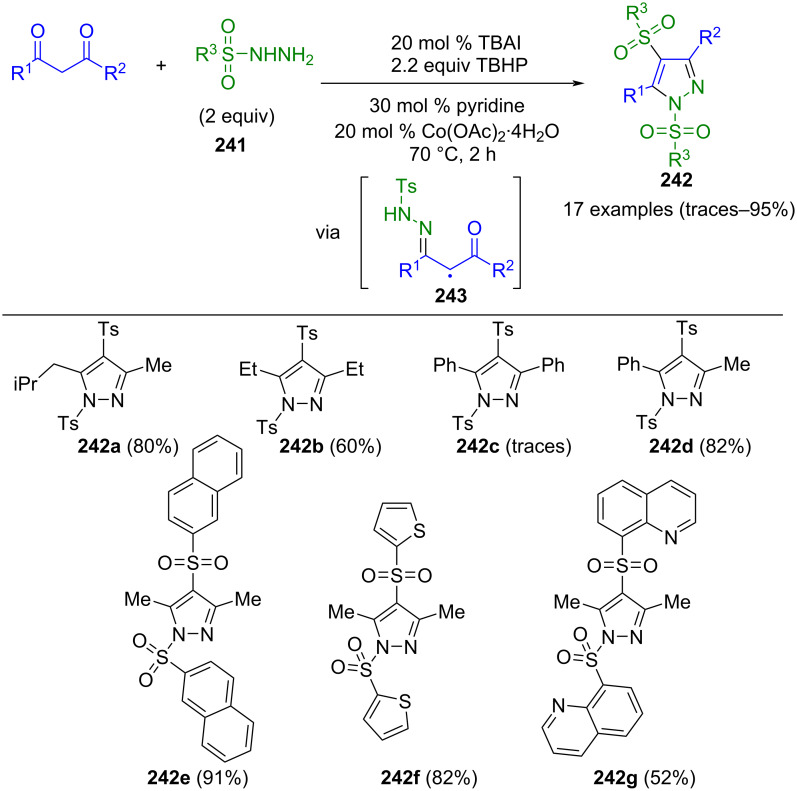
Pseudo-three-component synthesis of 1,4-bisulfonyl-substituted pyrazoles **242** [219].

In the synthesis of fully substituted pyrazoles by radical reactions, sulfonylhydrazines **241** can adopt a dual role. In a pseudo-three-component reaction involving 1,3-dicarbonyl compounds, along with tetra-*n*-butylammonium iodide (TBAI) as a catalyst and *tert*-butyl hydroperoxide (TBHP) as an oxidizing agent, these hydrazines serve both as a ring-forming component and as a sulfonyl precursor, to furnish 1,4-bisulfonyl substituted pyrazoles **242** (Scheme 85) [219]. Iodide causes the formation of *t-*BuO or *t-*BuOO radicals, which subsequently react with tosylhydrazine, liberating nitrogen nitrogen and generating a tosyl radical. H-Abstraction at the α-position of the resulting tosylhydrazone intermediate produces a further radical **243**. These radicals finally undergo cyclocondensation, resulting in the formation of the corresponding pyrazole **242**. Notably, this method tolerates many functional groups and can be scaled up to 16 grams. Only sterically demanding 1,3-dicarbonyl compounds and tosylhydrazines containing aliphatic residues do not lead to the desired product. Moreover, the reaction can be extended to enaminones [220].

**Scheme 86:**
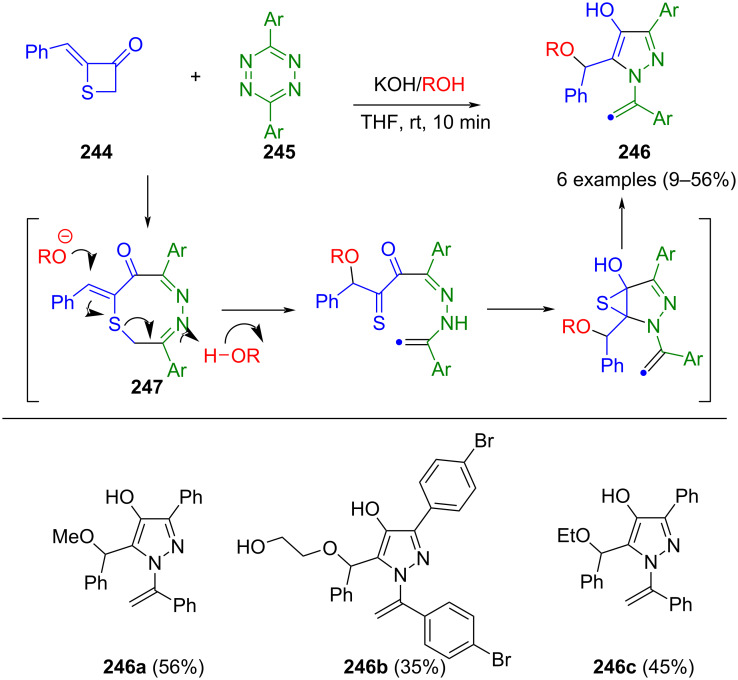
Three-component synthesis of 4-hydroxypyrazole **246** [221].

In an attempt to synthesize 2*H*-[1,4,5]thiadiazocin-7-ones **247** from thietanones **244** and 1,2,4,5-tetrazines **245**, Suen et al. made an interesting discovery. Although the desired product is formed in situ in this three-component sequence, the presence of alcohols under basic conditions triggers a condensation–fragmentation–cyclization–extrusion sequence to form fully substituted 4-hydroxypyrazoles **246** (Scheme 86) [221]. This allows for introducing an ether functionality. Notably, X-ray structural analysis shows that the isolated product is not the tautomeric pyrazolones.

## Conclusion

Pyrazoles are evergreens in heterocyclic chemistry, and the multitude of applications still demands for novel synthetic concepts for their preparation, particularly in a modular and diversity-oriented fashion for exploring structural and functional space. Multicomponent reactions (MCR) per se fulfill the claim for modularity and diversity-orientation. According to the reactivity-based principle of MCR, functionalities are created and consumed en route, minimizing the number of necessary steps. For MCR synthesis of pyrazoles, there are two key transformations: (3 + 2)-cyclocondensations and (3 + 2)-cycloadditions. Although α,β-unsaturated acceptors are relevant in both cases, in the former case, they rather become the C_3_ building block, while in the latter they serve as C2 blocks. This review delineates the major synthetic MCR strategies and highlights also the immediate potency of this approach, which is the concatenation of elementary organic and organometallic processes. The strategy not only allows extended modification but also exploiting reactivity patterns and establishing functional diversity for a variety of fields ranging from materials to life sciences. MCR establishes itself as a potent tool to uncover new properties of and propose new applications for pyrazoles due to the crucial synthetic expansion of the field.

## Data Availability

Data sharing is not applicable as no new data was generated or analyzed in this study.
